# Dual regulation of gastrointestinal tumor progression by the IFN-γ/STAT1 pathway and prospects for targeted therapy

**DOI:** 10.3389/fonc.2025.1598170

**Published:** 2025-08-20

**Authors:** Yuhao Liu, Yuxin Huang, Qiaoying He, Yuyan Shen, Yaoling Wang

**Affiliations:** School of Medicine, South China University of Technology, Guangzhou, Guangdong, China

**Keywords:** gastrointestinal tumors, the IFN-γ/STAT1 pathway, immunity, inflammation, tumor cell proliferation and metastasis, tumor cell death, precisely targeted therapy

## Abstract

Gastrointestinal malignant tumors exhibit a high incidence and mortality rate among all malignancies worldwide, making them a significant concern within the field of oncology. Targeted therapy for gastrointestinal tumors has become a hot topic in recent years, and its specific mechanism remains to be further elucidated. Secreted factors, including cytokines, chemokines, and growth factors, as components of the tumor microenvironment, play a crucial role in the progression of gastrointestinal tumors. Interferon-gamma (IFN-γ) can activate these factors through JAK1/2 and STAT1 signaling (the IFN-γ/STAT1 pathway). This pathway is considered “a double-edged sword” and maintains a dual role in promoting or inhibiting tumor progression by activating different downstream factors. In this review, we summarize the functions, mechanisms, and key factors of the IFN-γ/STAT1 pathway that promote or inhibit gastrointestinal tumor progression and discuss therapeutic prospects for targets of the pathway.

## 1 Introduction

Gastrointestinal malignant tumors continue to exhibit a high incidence and account for more than one-quarter of all malignancies and one-third of cancer-related deaths worldwide (1). Colorectal cancer (CRC) emerges as the most prevalent subtype, accounting for 38.5% of total gastrointestinal cancer cases, followed by stomach, liver, and esophageal malignancies (2, 3). Geographically, the highest incidence and mortality rates are observed in East Asia, particularly for gastric, liver, and esophageal cancers (2, 4). A recent analysis across 25 states in the United States indicated a notable rise in the incidence of colorectal, gallbladder, and pancreatic cancers among individuals aged 25 to 49 (5). Other studies have also shown that the incidence of pancreatic cancer, hepatobiliary cancer, and colorectal cancer is gradually increasing in people under the age of 50 (6, 7). These trends necessitate further investigation and intervention strategies to address the emerging patterns of gastrointestinal cancers in younger populations (8).

Currently, treatment options for gastrointestinal tumors include surgery, radiotherapy, and chemotherapy. However, the effectiveness of radiotherapy and chemotherapy varies due to differences in patient physical conditions and other factors, and they also carry potential adverse reactions. Despite numerous researchers’ significant contributions to gastrointestinal tumor therapies, achieving complete eradication of tumors and prolonged patient survival remains a formidable challenge.

The human digestive system consists of the digestive tract (which includes the oral cavity, pharynx, esophagus, stomach, small intestine, and large intestine) and the digestive glands (which include the salivary glands, pancreas, and liver). It develops from the endoderm and mesoderm during the development of the human embryo. Despite discrepancies in the organization, morphology, and biological functions of the digestive system’s organs, their shared origin and cooperation in the digestive and absorptive processes reveal commonalities. What they have in common indicates the potential for analogous mechanisms and the common expression of tumor-related factors in the progression of different gastrointestinal tumors (9, 10). Consequently, this also suggests the possibility of common therapeutic targets for gastrointestinal tumors.

Interferon-gamma (IFN-γ), a type II interferon, is predominantly produced by immune cells, including natural killer cells (NK cells), T helper 1 (T_H1_) cells, and CD8^+^ cytotoxic T lymphocytes (CTLs) (11). It plays a pivotal role in maintaining tissue homeostasis, mediating immune and inflammatory responses, and monitoring immunologic surveillance through the activation of downstream signaling by the IFN-γ receptor (12). The majority of cells express IFN-γ receptors and are regulated by IFN-γ. The IFN-γ signaling process is characterized by a cascade of tyrosine phosphorylation events triggered by the binding of IFN-γ to the IFN-γ receptor (IFN-γR), which leads to the initiation of the transcription of interferon-stimulated genes (ISGs). The IFN-γ/STAT1 signaling pathway represents the most classical IFN-γ signaling pathway. Upon binding of IFN-γ to its receptor, the IFN-γR1 chain and the IFN-γR2 chain oligomerize and transphosphorylate, activating downstream signaling components JAK1 and JAK2. Activated JAKs then phosphorylate the tyrosine residue at position 440 at the cytoplasmic terminus of IFN-γR1, resulting in its SH2 structural domain, thereby establishing the binding site of STAT1. The SH2 structural domain of STAT1 binds to the aforementioned site and forms homodimers of STAT1 after phosphorylation. The homodimers can enter the nucleus and bind to the γ-activation site (GAS) element in the promoter of ISGs to regulate the expression of downstream factors (13, 14).

The IFN-γ/STAT1 pathway plays a crucial role in regulating inflammation and immunity. Numerous studies have shown that its activation can act as a “double-edged sword” in the progression of human malignancies (15, 16). This phenomenon is also observed in gastrointestinal tumors, where various factors expressed downstream of the pathway can promote or inhibit tumor progression to varying degrees. Interestingly, the factors may have different effects depending on the specific site of different tumors or the stage of development of the same tumor. Therefore, it is essential to closely examine the functional similarities and heterogeneity of the IFN-γ/STAT1 pathway in gastrointestinal tumors when developing targeted therapeutic options focused on relevant downstream factors.

**Figure 1:**
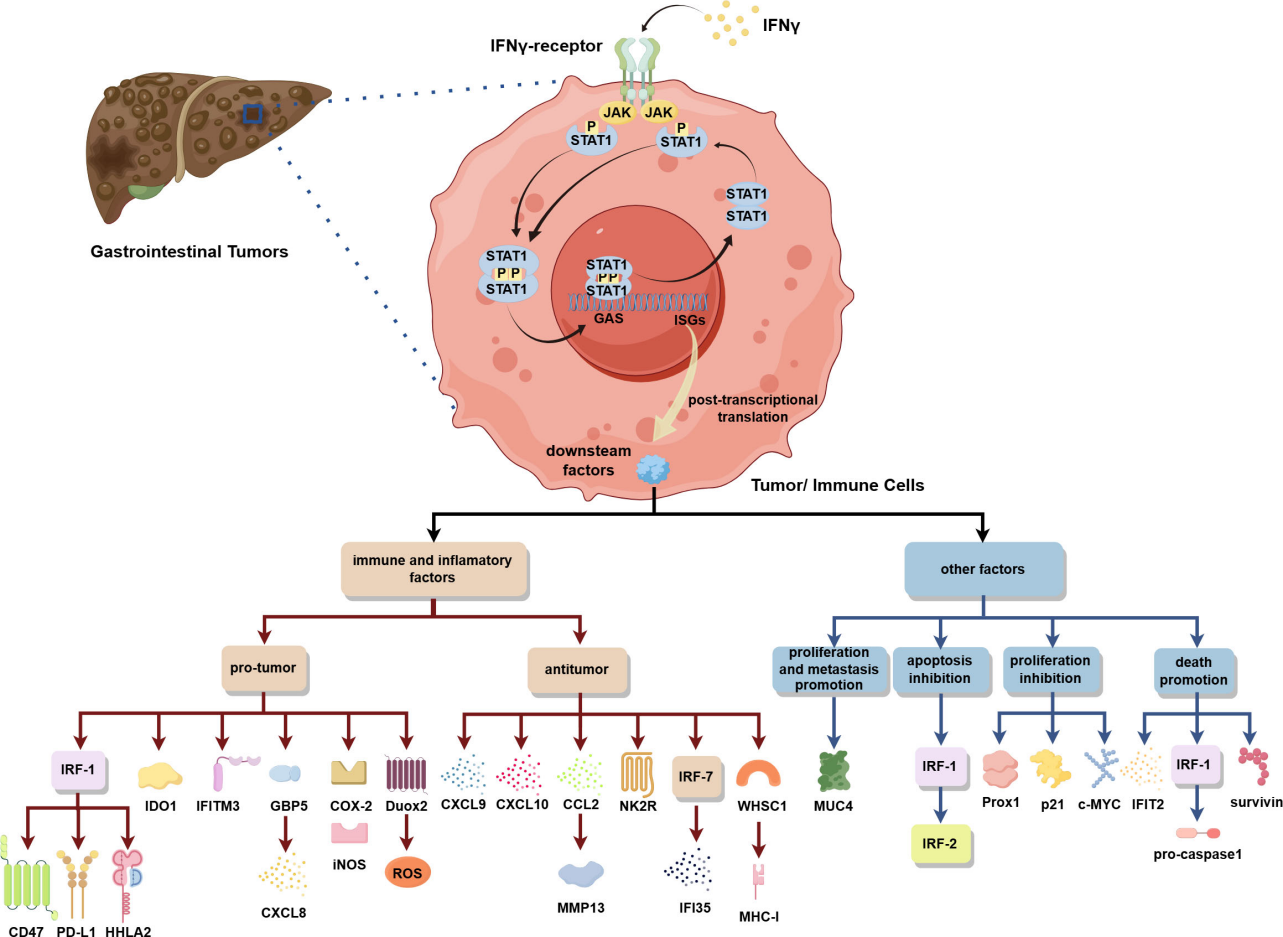
In gastrointestinal tumors, the IFN-γ/STAT1 pathway activation can regulate the expression of various downstream factors to play a pro-/antitumor role by influencing immunity, inflammatory environments, and tumor cell proliferation, metastasis, and death.

In this review, we summarized the research regarding the activation of the IFN-γ/STAT1 pathway in gastrointestinal tumors (**Figure 1**). We detailed the common and unique mechanisms by which the pathway promotes or inhibits tumors in different digestive organs, influencing antitumor immunity, tumorigenesis induced by inflammatory environments, tumor cell proliferation, metastasis, and death. This provides a more precise foundation for future targeted therapies. We also summarized the therapeutic molecules and medicines currently available for different parts of the pathway. Additionally, we summarized the current clinical treatment regimens for gastrointestinal tumors, discussed the development of immunotherapy, and explored promising immune-related therapeutic targets downstream of the pathway. We also proposed new directions for future clinical research.

## 2 Pathways that promote gastrointestinal tumor progression

Many cellular components and factors within the tumor microenvironment intricately regulate tumor pathogenesis (17). It is particularly evident in various gastrointestinal malignancies, where the composition of the tumor microenvironment and the relative proportions of distinct cell types demonstrate substantial heterogeneity (18). A pertinent example of this variability can be observed in the acid-base homeostasis of the gastric environment, which markedly contrasts with other organ systems. Such differences are likely to contribute to the variability in the expression of downstream factors associated with the IFN-γ/STAT1 pathway, which may lead to divergent effects—either promoting or inhibiting different gastrointestinal tumor progression.

**Figure 2:**
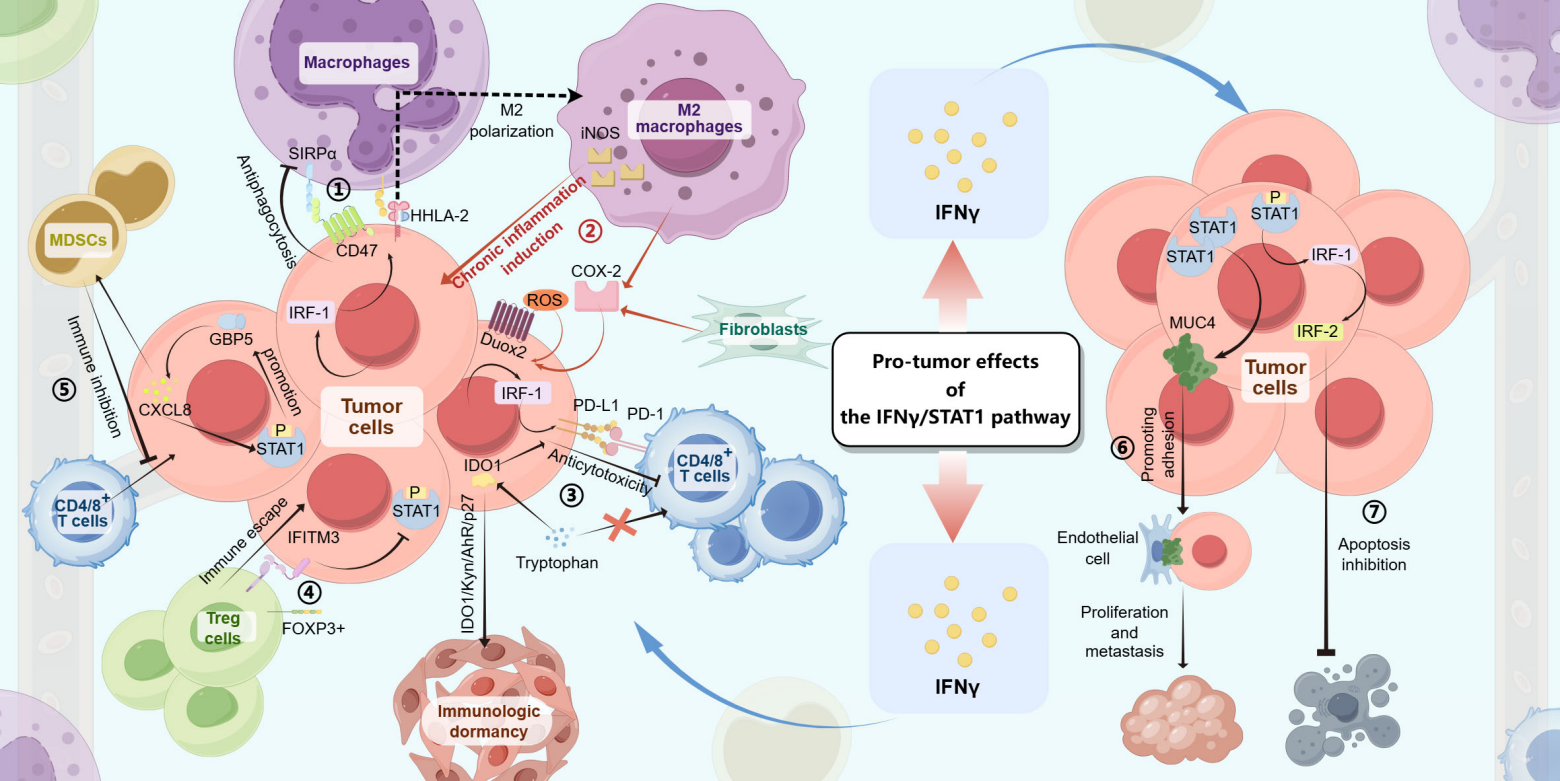
The IFN-γ/STAT1 pathway produces pro-tumor effects in gastrointestinal tumors through the following mechanisms: ① In tumor cells, the IFN-γ/JAK/STAT1 signaling induces IRF-1 expression, which then upregulates CD47 and HHLA-2 levels, resulting in antiphagocytosis and M2 polarization of macrophages. ② With the IFN-γ/JAK/STAT1 signaling, M2 macrophages, fibroblasts, and tumor cells produce downstream factors (iNOS, COX-2, and Duox2/ROS), making chronic inflammatory environments to induce tumorigenesis. ③ In tumor cells, both the IFN-γ/JAK/STAT1/IRF-1/PD-L1 pathway and the IFN-γ/JAK/STAT1/IDO1 pathway can lead to anticytotoxicity against CD4/8^+^ T cells by PD-L1 combining with PD-1 or competing with T cells for tryptophan. ④ The expression of IFITM3 induced by the IFN-γ/JAK/STAT1 signaling in tumor cells helps immune escape by interactions with Foxp3^+^ Treg cells and inhibits STAT1 phosphorylation-inducing apoptotic signaling. ⑤ The IFN-γ/JAK/STAT1/GBP5/CXCL8 feedback loop inhibit antitumor immune with the role of MDSCs, resulting in tumorigenesis. ⑥ The expression of MUC4 upregulated by the elevation of STAT1 levels promotes tumor cell proliferation and metastasis by the adhesion of tumor cells to endothelial cells. ⑦ The IFN-γ/JAK/STAT1/IRF-1/IRF-2 pathway inhibits tumor cell apoptosis.

Our study offers a comprehensive overview of the downstream effectors linked to the IFN-γ/STAT1 pathway, such as programmed death-ligand 1 (PD-L1), which have been extensively explored in tumors. These pathways are crucial to tumor progression as they facilitate immune escape, promote tumor proliferation and metastasis, inhibit apoptotic processes, and induce chronic inflammatory responses (**Figure 2**).

### 2.1 Pathways that promote immune escape

The immune system plays a critical role in inhibiting tumor progression through mechanisms of surveillance and clearance, primarily involving T cells, macrophages, and various other immune cells (19). However, the function of immune cells in mediating antitumor responses can be negatively regulated by specific immune checkpoints, such as the PD-1/PD-L1 checkpoint, as well as other factors associated with the tumor microenvironment. Consequently, immune escape is a significant mechanism underlying tumor progression *in vivo (* 20). The following studies have demonstrated that activation of the IFN-γ/STAT1 pathway enhances the expression of various downstream factors, facilitating tumor cell escape from immune-mediated destruction and contributing to disease progression.

#### 2.1.1 The IFN-γ/JAK/STAT1/IRF-1/downstream pro-tumor factor pathways

The interferon regulatory factor (IRF) family is prominently recognized for its pivotal role in the modulation of gene expression underlying the interferon response. This family comprises nine members (IRF-1 to IRF-9) in humans and mice, each having distinct functions in regulating immune responses and tumorigenesis. Numerous investigations into gastrointestinal tumors have revealed that IRF-1 exhibits a ‘double-edged sword’ role, simultaneously promoting and inhibiting tumor progression by regulating various downstream factors mediated by the IFN-γ/STAT1 pathway. As one of the most extensively studied members of the IRF family, IRF-1 has been demonstrated to influence the differentiation of immune cell subsets, including T cells, and to suppress oncogene transcription and expression (21). Conversely, it may also facilitate the progression of gastrointestinal tumors by modulating critical effector molecules such as CD47 and PD-L1.

CD47 is a glycoprotein featuring five transmembrane domains widely expressed in normal and tumorous cells (22). It interacts with SIRPα receptors on phagocytes, such as macrophages, to convey a ‘don’t-eat-me’ signal, thereby preventing cell death through phagocyte engulfment and contributing to tumor immune escape (23). A study involving the tumor cell line HCT116 (human colorectal cancer) has shown that CD47 regulation is influenced by IFN-γ through the action of STAT1-mediated IRF-1, leading to an upregulation of CD47 expression in tumor cells. This upregulation increases the binding affinity of CD47 for SIRPα, consequently diminishing macrophage-mediated phagocytosis of tumor cells and facilitating immune escape (24). However, additional experimental validation is necessary to fully understand the impact of IFN-γ on immune escape in colorectal tumors *in vivo*.

PD-L1, also known as B7-H1 and identified by the nomenclature CD279, is a member of the B7 family of proteins. Its expression is primarily induced by pro-inflammatory mediators and is observed in macrophages, activated T and B lymphocytes, dendritic cells (DCs), and specific epithelial cells (25, 26). Notably, PD-L1 is also expressed in various tumor cells. PD-1 is a transmembrane protein in activated T and B lymphocytes, NK cells, macrophages, DCs, and monocytes (27). PD-1 and PD-L1 function as immune checkpoints, critical in regulating immune tolerance within the tumor microenvironment and facilitating immune escape by tumors through inhibiting T cell activation, proliferation, and cytotoxicity. Recent studies have shown that PD-L1 expression in tumor cells, particularly in gastrointestinal malignancies such as oral squamous cell carcinoma, esophageal squamous cell carcinoma, gastric cancer, pancreatic cancer, liver cancer, and colorectal cancer, is regulated via the IFN-γ/STAT1 pathway, with IRF-1 acting as a key downstream mediator. Activation of the IFN-γ/STAT1 signaling promotes the transcription of IRF-1, upregulating PD-L1 expression in tumor cells. This upregulation of PD-L1 facilitates its interaction with PD-1 on immune cells, including CD4/8^+^ T cells, thereby contributing to the immune escape associated with gastrointestinal tumors (28–34). Furthermore, the unique characteristics of the tumor microenvironment also influence PD-L1 expression. A study involving colorectal cancer cells has shown that the acidosis typical of the tumor microenvironment increases PD-L1 expression in the presence of IFN-γ *in vitro*. This enhancement appears to be associated with elevated expression and phosphorylation of STAT1 (35). Nevertheless, the potential for this phenomenon *in vivo* remains to be established. Further research is warranted to explore the hypothesis that the acid environment may promote immune escape in gastrointestinal tumors via the PD-1/PD-L1 immune checkpoint, considering the distinct acid-base environments across different organs.

A recent investigation into liver cancer has elucidated the role of IRF-1 in regulating the downstream factor HHLA2, facilitating immune escape, and promoting tumor progression. This phenomenon is attributed to the expression of HHLA2 in liver cancer cells, which induces M2 polarization and the chemotactic migration of macrophages. The resulting phenotype is characterized by increased tumor-associated macrophages, known for their immunosuppressive properties and upregulation of PD-L1 expression in tumor cells. This process demonstrates a dose-dependent relationship with IFN-γ (36). Notably, while this study does not directly establish that IFN-γ exerts its regulatory effects on HHLA2 expression via a STAT1-mediated mechanism, the existing research addressing the interplay between IFN-γ and IRF-1 provides us a compelling foundation for hypothesizing the involvement of the IFN-γ/JAK/STAT1/IRF-1/HHLA2 pathway in the promotion of tumor progression through the facilitation of immune escape in liver cancer. Subsequent experimental investigations will be essential to ascertain whether STAT1 mediates this intricate process.

In addition to regulating the expression of immune escape-related factors, IRF-1 can also act as an oncogenic protein in gastrointestinal tumors. Notably, low levels of IRF-1 expression have been identified in liver cancer. Furthermore, IRF-1 can inhibit downstream factors such as ZEB1, which may help suppress the epithelial-mesenchymal transition, migration, and invasion of tumor cells (37). In summary, IRF-1 plays a multifaceted role in the progression of gastrointestinal tumors, primarily through the IFN-γ/STAT1 pathway.

#### 2.1.2 The IFN-γ/JAK/STAT1/IDO1 pathway

Indoleamine-2,3-dioxygenase 1 (IDO1) functions as a critical intracellular enzyme and serves as a rate-limiting factor in the metabolism of tryptophan (Trp) within the kynurenine (Kyn) pathway. Its activity can result in the depletion of tryptophan in the tumor microenvironment, subsequently inhibiting T cell proliferation and function, thus facilitating the immune escape of neoplastic cells (38). Research indicates that in liver cancer, IDO1 expression is upregulated by IFN-γ through the JAK1/STAT1 signaling, which undermines T cell functionality, aiding in the immune escape of tumors (39). Furthermore, evidence from studies conducted on the tumor cell lines H22 (hepatocellular carcinoma) and CT26 (colon cancer) suggests that the IFN-γ/JAK/STAT1/IDO1 pathway not only aids tumor cells in immune escape by affecting T cell function but also inhibits tumor cell apoptosis by inducing tumor-repopulating cells to enter immunologic dormancy through a downstream signaling mechanism involving IDO1/Kyn/AhR/p27 pathway (40). Additionally, IDO1-expressing Paneth cells, influenced by STAT1 in colorectal cancer, can promote immune escape by modulating the tumor load through immune cell infiltration (41). Thus, the activation of the IFN-γ/JAK/STAT1/IDO1 pathway contributes to immune escape in gastrointestinal tumors via its effects on various cell types, including T cells and tumor cells. This result also suggests that the combination of IFN-γ and IDO/AhR inhibitors may be a potentially effective immunotherapeutic modality.

#### 2.1.3 The IFN-γ/JAK/STAT1/IFITM3 feedback loop

Interferon-induced transmembrane protein 3 (IFITM3) is an antiviral effector protein that is upregulated by interferon cytokines and has been demonstrated to inhibit cell infection by a broad spectrum of viruses. Furthermore, IFITM3 enhances the functionality of resident memory CD8^+^ T cells, which are known for their robust antiviral resistance (42, 43). Notably, IFITM3 is frequently overexpressed in various tumor cells, correlating with the histopathological grading and staging of tumors (44). However, the underlying mechanisms remain inadequately understood. In colorectal cancer, research indicates that IFITM3 expression is modulated via the IFN-γ/STAT1 pathway, which plays a significant role in immune escape by influencing the stability of regulatory T (Treg) cells and the maintenance of immune homeostasis. FOXP3^+^ Treg cells have been shown to suppress antitumor immunity, whereas impaired IFITM3 functionality disrupts this inhibitory effect, resulting in heightened IFN-γ expression and an augmented antitumor immune response. Thus, IFITM3 is recognized as a critical factor in the Treg-mediated immune escape mechanisms. Interestingly, IFITM3 also exhibits feedback inhibition on the phosphorylation and nuclear translocation of STAT1, regulating the autophagy-mediated degradation of STAT1 to mitigate IFN-γ secretion. This interplay establishes an IFN-γ-dependent feedback loop between STAT1 and IFITM3 essential for preserving Treg cell function, attenuating antitumor immunity, and facilitating immune escape (45). Thus, targeting inhibition of IFITM3 and breaking this negative feedback loop for IFN-γ-mediated antitumor immunosuppression could be an option to modulate the tumor immune microenvironment in gastrointestinal tumors for therapies.

#### 2.1.4 The IFN-γ/JAK/STAT1/GBP5/CXCL8 feedback loop

GBP5, a member of the translational factor class of the dynamin-like GTPase superfamily (46), has been implicated in malignancy-associated functions in the progression of various tumors, including glioblastomas and breast cancers (47, 48). Recent studies have identified GBP5 as a pivotal cytokine involved in the assembly of inflammatory vesicles, serving as a central coordinator of immune responses to oncological diseases. Its clinical significance is underscored by its potential implications for prognosis (49, 50). Research highlights that GBP5 is markedly upregulated in gastric cancer, which is mediated by the IFN-γ/STAT1 signaling. This phenomenon promotes the proliferation and migration of gastric cancer cells. Beyond its direct regulatory effects on gastric tumors, GBP5 is known to induce the expression of CXCL8. This chemokine can infiltrate the tumor microenvironment by recruiting myeloid-derived suppressor cells (MDSCs), facilitating immune escape. Notably, CXCL8 exhibits dual functionality. It exerts immunosuppressive effects and enhances JAK1/STAT1 signaling, leading to an increased expression of GBP5. This interaction establishes a positive feedback mechanism characterized by the IFN-γ/JAK/STAT1/GBP5/CXCL8 loop, promoting tumor cell proliferation, invasion, and immune escape (51). Breaking the persistent immune escape mediated by the positive feedback loop is the key to targeted therapies against GBP5 and CXCL8.

### 2.2 Pathways promote tumor progression by mediating chronic inflammation

Inflammation is recognized as a significant precursor to the development of tumors, with research indicating that approximately 20% of malignancies may be initiated by inflammatory processes (52). Numerous studies have reported that chronic pancreatitis can lead to the transformation of pancreatic tissues into pancreatic cancer through acinar-ductal metaplasia (53). The IFN-γ/STAT1 pathway has been identified as a crucial mediator of inflammation, facilitating tumorigenesis in the digestive system by activating a range of ISGs. The involvement of the pathway in the pathophysiology of pancreatic and colorectal cancers has been extensively documented in the literature.

#### 2.2.1 The IFN-γ/JAK/STAT1/COX-2, iNOS pathways

Inducible nitric oxide synthase (iNOS) catalyzes the production of nitric oxide (NO) in significant quantities, particularly within macrophages. It is closely linked to the inflammatory responses observed in various organs. This mechanism plays a critical role in regulating immune diseases (54). Cyclooxygenase-2 (COX-2) is released into the tumor microenvironment by cancer-associated fibroblasts, M2 macrophages, and tumor cells, facilitating the induction of cancer stem cell-like activities. Furthermore, COX-2 promotes cell proliferation, angiogenesis, inflammation, invasion, and metastasis of tumor cells (55). Evidence from relevant studies has demonstrated that producing COX-2 and iNOS, downstream effectors of the IFN-γ pathway (56, 57), is crucial for developing inflammation-mediated colorectal tumors (58, 59). Notably, iNOS and COX-2 exhibited significantly elevated expression levels in tissues expressing IFN-γ compared to those lacking IFN-γ expression, with a corresponding increase in STAT1 phosphorylation. This finding suggests that COX-2 and iNOS may function as downstream mediators of STAT1 activity. In colorectal cancer, COX-2 is prominently detected in macrophages within non-tumor regions and in tumor-associated macrophages and tumor cells. In contrast, iNOS expression in tumor cells is characterized by weak signaling within the tumor microenvironment (60). These observations imply that COX-2 may play a multifaceted role in tumor progression by inducing IFN-γ across various cell types throughout tumor progression. In contrast, iNOS appears to be primarily associated with macrophage activity. This distinction underscores the differential roles of the IFN-γ/STAT1 pathway across various cellular contexts. The mechanisms by which iNOS and COX-2 contribute to tumorigenesis within the digestive system may entail inflammatory injury, KRAS gene mutations, and aberrant cell proliferation (53). These findings provide a basis for the timely elimination of the inflammatory environment in gastrointestinal tissues to prevent tumorigenesis.

#### 2.2.2 The IFN-γ/JAK/STAT1/Duox2/ROS pathway

Dioxygenase 2(Duox2), a NADPH oxidase gene family member, functions as a membrane glycoprotein. Variants of Duox2 have been associated with disturbances in microbiota immune homeostasis and a heightened susceptibility to inflammatory bowel disease. Dioxygenase A2(DuoxA2), located within the endoplasmic reticulum, is integral in modulating the enzymatic activity of Duox2 (61). Both Duox2 and DuoxA2 exhibit overexpression in human pancreatic and colorectal cancer cells, thereby increasing the susceptibility of these cells to tumor progression via the generation of elevated levels of reactive oxygen species (ROS) that promote tumor progression. In pancreatic and colorectal cancer cells, the expression of Duox2 is regulated by the transcription factor STAT1. Notably, the production of ROS and H2O2, reliant on the activity of the Duox2/DuoxA2 complex, displays significant upregulation in cells subjected to treatment with IFN-γ, influencing both intracellular and extracellular environments. This increase in reactive species may contribute to a pro-inflammatory microenvironment within the pancreas and colorectum, facilitating tumorigenesis and progression. Furthermore, the IFN-γ/JAK/STAT1/Duox2/ROS pathway has been implicated in enhancing genomic instability and compromising the functionality of oncogenes, including serine/threonine and tyrosine phosphatases, which are pivotal in regulating the proliferation of transformed cells (62). Moreover, Duox2 has been identified as a contributor to establishing a pro-angiogenic extracellular environment, which may further promote tumor growth and leukocyte infiltration (52). Targeting Doux2 will enable the therapy of gastrointestinal tumors in terms of the inflammatory environment and tumor growth.

### 2.3 Pathways promote tumor progression by promoting tumor cell proliferation and metastasis

The proliferation of tumor cells and their metastatic dissemination are crucial determinants in the pathogenesis and progression of tumors *in vivo*. Exploring the factors that promote tumor cell proliferation and metastasis is imperative while elucidating the underlying mechanisms involved. Numerous studies have demonstrated that the activation of the IFN-γ/STAT1 pathway significantly improves the expression of several downstream factors, contributing to tumor cell proliferation and metastasis. This activation ultimately facilitates the progression of gastrointestinal malignancies.

#### 2.3.1 The IFN-γ/JAK/STAT1/MUC4 pathway

MUC4, a transmembrane mucin, is critical in protecting and lubricating epithelial cells and is integral to cell renewal and differentiation (63). MUC4 has been implicated in promoting tumor progression through various mechanisms. These include inhibiting apoptosis, promoting proliferation and invasion, and indirectly modulating interactions between neoplastic cells and extracellular matrix proteins (64, 65). Under pathological conditions, particularly in pancreatic cancer, MUC4 is markedly overexpressed and contributes to tumorigenesis by facilitating the adhesion of tumor cells to endothelial cells and promoting metastasis (66–70). Research has elucidated the regulatory role of the IFN-γ/JAK/STAT1 pathway in modulating MUC4 expression within pancreatic cancer cells. Notably, IFN-γ evokes MUC4 expression in the cell line CD18/HPAF-SF (pancreatic adenocarcinoma) in a dose- and time-dependent manner. An analysis of expression levels reveals a significant temporal increase in MUC4 concurrent with the upregulation of STAT1. However, the modulation of MUC4 expression is characterized by a relatively delayed response compared to the rapid induction of IRF-1, primarily dependent on the immediate activation of phosphorylated STAT1 (pY701-STAT1) (71). Further inquiry has indicated that the elevation of STAT1 levels, independent of its phosphorylation status, is a pivotal determinant for the induction of MUC4 (72). This idea suggests a distinct regulatory pathway for MUC4 that diverges from the conventional JAK/STAT1 pathway characterized by IRF-1 gene activation. It is hypothesized that the accumulation of non-tyrosine-phosphorylated STAT1 in the nucleus and its interaction with GAS elements may play a crucial role. This newly identified regulatory framework complicates the understanding of gene regulation in gastrointestinal tumors. Despite the consistent upstream activation of the IFN-γ/STAT1 pathway, a noteworthy heterogeneity persists in activating specific downstream targets across various organs. In summary, modulation of MUC4 expression via the IFN-γ/JAK/STAT1 pathway significantly promotes pancreatic cancer cell proliferation and metastasis.

### 2.4 Pathways promote tumor progression by inhibiting tumor cell apoptosis

Apoptosis is a crucial component of the cellular life cycle, functioning as a regulatory mechanism for cellular turnover and homeostasis. Tumor cells can proliferate indefinitely, primarily due to the dysregulation of normal apoptotic processes. While the body of literature on this subject remains somewhat limited, emerging studies indicate that the activation of the IFN-γ/STAT1 pathway in gastrointestinal malignancies initiates the expression of downstream factors that inhibit apoptotic pathways. This inhibition can facilitate the progression of gastrointestinal tumors, highlighting a critical area of investigation for potential therapeutic intervention.

#### 2.4.1 The IFN-γ/JAK/STAT1/IRF-1/IRF-2 pathway

The role of IRF-1, a prominent member of the IRF family, in facilitating tumorigenesis within the gastrointestinal system through immune escape mechanisms has been previously elucidated in the literature. In conjunction with modulatory factors such as IRF-2, it serves as a target of regulation by IRF-1. Its expression exerts inhibitory effects on the IRF-1-mediated transcriptional regulation of various downstream genes (73). Notably, a study focusing on esophageal cancer cells revealed that the expression of IRF-2 was significantly upregulated in response to low concentrations of IFN-γ, contributing to the pathogenesis of esophageal cancer. The underlying mechanism involves the ability of IRF-2 to bind to specific sequences within the promoter region of IFN-γR1, subsequently attenuating the expression of IFN-γR. This reduction in receptor expression leads to decreased sensitivity of esophageal cancer cells to IFN-γ, thereby mitigating the apoptosis typically induced by IFN-γ in the context of cancer cell interactions (74). Consequently, this negative feedback loop facilitates tumor progression by impairing the apoptotic response elicited by IFN-γ in esophageal cancer cells.

## 3 Pathways that inhibit gastrointestinal tumor progression

**Figure 3:**
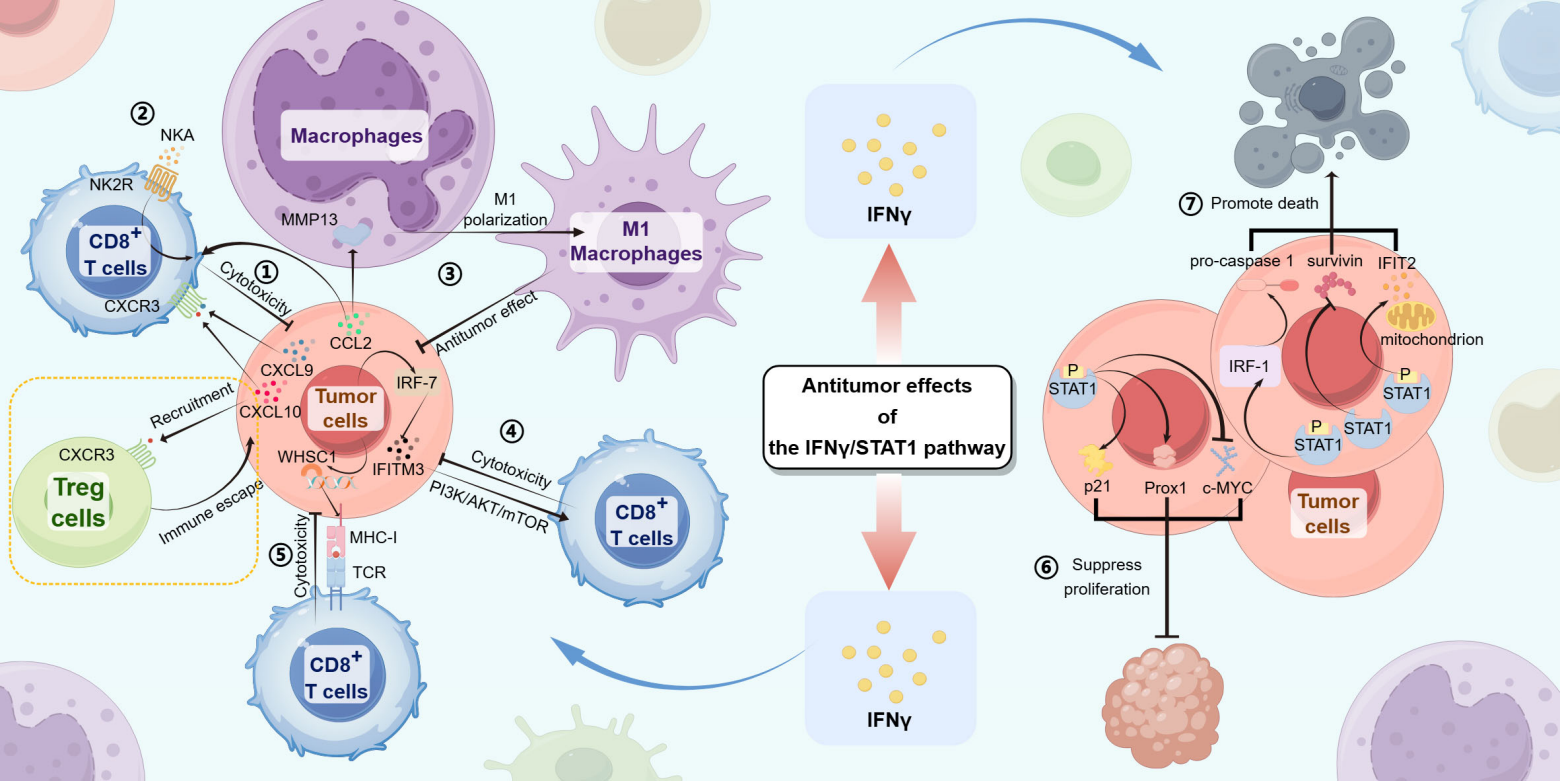
The IFN-γ/STAT1 pathway produces antitumor effects in gastrointestinal tumors through the following mechanisms:① In tumor cells, the IFN-γ/JAK/STAT1 signaling induces CXCL9 and CXCL10 expression, promoting cytotoxicity from CD8^+^ T cells by the interactions between CXCL9/10 and CXCR3. ② With the IFN-γ/JAK/STAT1 signaling, CD8+ T cells upregulate NK2R expression, combining with NKA to promote cytotoxicity from CD8^+^ T cells. ③ Tumor cells express CCL2 that not only induces MMP13, resulting in M1 polarization of macrophages but also actives CD8+ T cell cytotoxicity, promoting antitumor effects by the IFN-γ/JAK/STAT1 signaling. ④ The IFN-γ/JAK/STAT1/IRF-7/IFITM3 pathway actives the downstream PI3K/AKT/mTOR signaling, inhibiting tumor progression by CD8^+^ T cell cytotoxicity. ⑤ WHSC1, induced by the IFN-γ/JAK/STAT1 signaling in tumor cells, upregulates the expression of MHC-I, promoting combination with TCR and CD8+ T cell cytotoxicity. ⑥ The upregulated expression of Prox1 and p21 and the downregulated expression of c-MYC in tumor cells both lead to the suppression of tumor cell proliferation. ⑦ The upregulated expression of IFIT2 and IRF-1/pro-caspase-1 leads to tumor cell death. STAT1 directly inhibits survivin, leading to tumor cell apoptosis.

The preceding section provided a detailed examination of the role of the IFN-γ/STAT1 pathway in promoting gastrointestinal tumors. This discussion highlighted four principal elements: the facilitation of immune escape, inflammation-associated tumorigenesis, direct promotion of tumor growth and metastasis, and the inhibition of tumor apoptosis. The following section summarizes the inhibitory effects exerted by the IFN-γ/STAT1 pathway on the progression of gastrointestinal tumors. It emphasizes three critical dimensions: the enhancement of anti-tumor immunity, the suppression of tumor cell proliferation, and the promotion of tumor cell death (**Figure 3**).

### 3.1 Pathways inhibit tumor progression by enhancing antitumor immunity

The IFN-γ/STAT1 pathway’s involvement in facilitating tumor progression has been previously discussed. Beyond the pro-tumor targets associated with the pathway, several additional targets are linked to fostering antitumor immune responses, such as the CXCL chemokine family and NK2R receptor. These factors enhance the immune activity of effector cells, particularly CD8^+^ T cells, thereby playing a pivotal role in suppressing gastrointestinal tumor progression.

#### 3.1.1 The IFN-γ/JAK/STAT1/CXCL chemokine family pathways

The CXCL chemokine family represents an integral class of signaling factors, predominantly secreted by tumor cells, leukocytes, and a variety of other cellular entities, and is pivotal in regulating a myriad of biological processes, including tumor progression and immune homeostasis. This family encompasses approximately 20 distinct members, each characterized by specific functions manifesting in particular physiological contexts (75). Notably, CXCL9, CXCL10, and CXCL11 utilize the shared receptor CXCR3 and engage in similar mechanisms that facilitate the recruitment of T cells and other immune cells to the tumor microenvironment, contributing to antitumor responsiveness. Recent investigations have elucidated that CXCL11 is regulated predominantly by STAT2, as opposed to STAT1, within the skin and in various related pathologies (76). The current research on gastrointestinal tumors has further validated the critical role of the IFN-γ/STAT1/JAK/CXCL chemokine pathway—particularly involving CXCL9 and CXCL10—in promoting antitumor immune responses. Such findings underscore the importance of these chemokines in orchestrating immune surveillance and therapeutic strategies against tumors.

CXCL9, belonging to the CXCL chemokine family, is predominantly induced by IFN-γ rather than interferon-alpha/beta (IFN-α/β) (77). The prevailing theory suggests that CXCL9 is crucial in mediating lymphocyte infiltration into specific tumor sites while concurrently inhibiting tumor progression. This relationship is exemplified by the IFN-γ/STAT1/CXCL9 pathway, which has been validated in multiple studies (78). Recent research utilizing murine lung and colorectal cancer cell lines reveals that Cyclin G2, a specific protein, is integral to the expression of the macrophage IFN-γ/JAK/STAT1/CXCL9 pathway. This pathway enhances the anticarcinogenic efficacy of immune cells, particularly CD8+ T cells, as Cyclin G2 facilitates the nuclear translocation of STAT1 through its interaction with PP2Ac, thereby increasing CXCL9 expression. Further investigations are warranted to ascertain whether similar mechanisms are operational in other gastrointestinal malignancies (79). Notably, in the context of squamous cell carcinoma of the tongue, CXCL9 has been demonstrated to interact directly with tumor cells via its receptor CXCR3, thereby promoting tumor invasion and metastasis (80). However, it remains ambiguous whether the regulation of this interaction by the IFN-γ/STAT1 pathway is involved. Should this regulation be confirmed, the underlying reasons for the observed disparities in the effects of CXCL9 across various gastrointestinal tumors warrant further exploration.

CXCL10, another member of the CXCL family, is recognized as an IFN-γ-inducible protein (IP-10). Unlike CXCL9, CXCL10 can be weakly induced by TNFα in addition to being strongly induced by all three of IFN-γ, IFN-α, and IFN-β (81). Studies have illustrated that CXCL10 plays a significant role during the early phases of hypoxia-induced inflammation, with its expression correlating positively with lymphocytic infiltration during tumor therapies (82, 83). Recent findings have elucidated that in gastric glandular cancer, regulated by IFN-γ/STAT1 signaling, the upregulation of CXCL10 correlates strongly with the degree of CD8^+^ T cell infiltration in the tumor microenvironment, thereby bolstering antitumor immunity. This phenomenon is closely associated with the expression levels of PML in gastric glandular cancer cells (84). Interestingly, it has been demonstrated that the signaling of CXCR3 by CXCL10 plays a direct role in the recruitment of Treg cells after liver transplantation in patients with liver cancer and hepatic tumor recurrence (85). Some studies have suggested that this may be related to the mobilization of response to endothelial progenitor cells (86). It remains unclear whether the IFN-γ/STAT1 signaling regulates the immune escape for tumor progression demonstrated here by CXCL10/CXCR3.

#### 3.1.2 The IFN-γ/JAK/STAT1/CCL2/MMP13 pathway

CCL2, or monocyte chemoattractant protein-1 (MCP-1), is synthesized by various cell types, including tumor and endothelial cells (87). It plays a pivotal role in regulating the infiltration and migration of monocytes, NK cells, and other immune cells, contributing significantly to the immune response (88). MMP13, a member of the matrix metalloproteinase (MMP) family, is involved in the degradation of various extracellular matrix components and is crucial to tissue remodeling, inflammatory responses, and tumor progression. It has been implicated in tumor infiltration and angiogenesis during photocarcinogenesis, thereby contributing to the invasive characteristics of melanomas and other malignancies (89). Investigations into pancreatic cancer have revealed that both CCL2 and MMP13 are regulated by the upstream IFN-γ/STAT1 signaling, which facilitates macrophage polarization. Notably, this polarization manifests more in M1-type macrophages, enhancing their antitumor activity and inhibiting tumor progression. This molecular mechanism has been validated *in vitro* using the RAW 264.7 mouse monocyte/macrophage-like cell line and in the KPC mouse model. Furthermore, CCL2 expression has been shown to drive T cell recruitment, thereby exerting appreciable antitumor effects, likely due to its capacity to mediate T cell attraction and activation (90). In conclusion, this study elucidates the role of the IFN-γ/JAK/STAT1/CCL2/MMP13 pathway in exerting antitumor effects in pancreatic cancer by activating immune cells, which challenges the conventional understanding of CCL2 and MMP13 as promoters of tumor progression. Future research is warranted to determine whether this mechanism can be extrapolated to other gastrointestinal tumors.

#### 3.1.3 The IFN-γ/JAK/STAT1/NK2R pathway

Neurokinin A (NKA) is an excitatory neurotransmitter in the central and peripheral nervous systems (91). Its receptor, NK2R, binds to NKA and initiates various downstream signaling pathways. Recent research has expanded our understanding of the role of NKA beyond the nervous system, particularly in gastrointestinal tumors, where it appears to have a significant function related to the IFN-γ/STAT1 pathway (92–94). For instance, a study using a mouse model of liver cancer demonstrated that IFN-γ enhances NK2R expression in CD8^+^ T cells, which boosts their ability to kill tumor cells (95). This enhancement is dependent on STAT1 signaling. The mechanism involves the upregulation of NK2R, which increases NKA binding and activates CD8^+^ T cells, facilitating the phosphorylation of ERK1/2 and the activation of the nuclear factor-κB (NF-κB) signaling pathway. These processes are vital for the activation, proliferation, and cytotoxic functionality of CD8^+^ T cells (96). Thus, the IFN-γ/JAK/STAT1/NK2R pathway is critical in inhibiting the advancement of liver cancer by enhancing the antitumor immune response through CD8^+^ T cell cytotoxicity. Moreover, a study suggests that IFN-γ promotes the expression of both NKA and NK2R in dendritic cells via a STAT1-dependent mechanism. This elevated NK2R expression strengthens NKA signaling and further stimulates the development of specific CD4^+^ and CD8^+^ T cells (95). More research is needed to determine whether this relationship holds across gastrointestinal tumors.

#### 3.1.4 The IFN-γ/JAK/STAT1/IRF-7/IFI35 pathway

Interferon-inducible protein 35 kDa (IFI35) is expressed in diverse cells and significantly modulates immune-inflammatory responses across various tissues (97–99). Recent investigations have suggested that IFI35 may possess antitumor properties, and this idea is now confirmed in colorectal cancers. Evidence indicates that the expression of IFI35 in murine colorectal cancer cells is regulated by IFN-γ, with dependencies on STAT1 and IRF-7, thus establishing the IFN-γ/JAK/STAT1/IRF7/IFI35 pathway. Subsequent studies have illustrated that CD8^+^ T cells, upon activation through IFI35 expressed by tumor cells, stimulate the PI3K/AKT/mTOR pathway, which enhances cell proliferation and the production of cytotoxic effector molecules, thereby augmenting the immune-mediated cytotoxicity against tumor cells. Notably, activating the pathway reinforces the antitumor capabilities of CD8^+^ T cells and improves the efficacy of CAR-T cell therapies (100). However, it is imperative to note that the IFN-γ/JAK/STAT1/IRF-7/IFI35 pathway, as characterized in this study, has yet to be directly validated *in vivo* within a mouse model of colorectal cancer. There is currently a lack of reports demonstrating analogous effects in other gastrointestinal tumors, indicating an area that warrants further investigation.

#### 3.1.5 The IFN-γ/JAK/STAT1/WHSC1/MHC-I pathway

IFN-γ–stimulated MHC class I (MHC-I) antigen presentation underlies the core of antitumor immunity (101). Histone dimethyltransferase WHSC1 is a SET domain-containing histone methyltransferase that catalyzes the dimethylation of lysine 36 of histone H3 (H3K36me2), a mark associated with actively transcribed genes (102, 103). WHSC1 is either overexpressed or hyperactivated in multiple myeloma, acute lymphoblastic leukemia, and prostate tumors, resulting in increased methylation of H3K36 on promoters of oncogenes (104, 105). However, a study in colorectal cancer cells has shown that the IFN-γ/STAT1 signaling promotes MHC-I gene expression by upregulating the expression of WHSC1, which interacts with NLRC5, enhancing H3K36me2 modifications of MHC-I genes. This phenomenon consequently strengthens the antitumor immunity via the IFN-γ/STAT1 pathway in gastrointestinal tumors (101). Furthermore, the multiple roles of the pathway are revealed to us from the new perspective of epigenetics. Perhaps regulating epigenetic modifications of tumor-associated genes also serves as a new strategy for gastrointestinal tumor therapies.

### 3.2 Pathways inhibit tumor progression by suppressing tumor cell proliferation

Suppression of tumor cell proliferation constitutes a fundamental approach to thwarting tumorigenesis and its progression. Therefore, elucidating the molecular mechanisms underpinning the inhibitory effects of the IFN-γ/STAT1 pathway on tumor progression is of paramount significance for advancing therapeutic strategies in oncology. The subsequent section will systematically delineate relevant molecular pathways that suppress tumor cell proliferation in gastrointestinal tumors.

#### 3.2.1 The IFN-γ/JAK/STAT1/Prox1 pathway

Prox1, a transcription factor that has been evolutionarily conserved, belongs to the family of homeodomain-containing transcription factors (106). This protein is integral to the development and differentiation processes of various tissues and organs (107). Prox1 is expressed in many malignancies, and its involvement in tumorigenesis and tumor dissemination has been posited. Observations indicate a marked decrease in Prox1 expression across several gastrointestinal tumors, like liver cancer and pancreatic cancer, with the degree of reduction correlating significantly with the differentiation status of the tumor (108–110). Overexpression of Prox1 in tumor cells has been shown to inhibit both proliferative and transformational activities (111). This finding was also validated in esophageal cancer, where IFN-γ induced Prox1 by activating STAT1 in esophageal squamous cell carcinoma (ESCC). Furthermore, overexpression of Prox1 was found to inhibit tumor cell proliferation in ESCC, demonstrating its role as an antitumor factor (112). However, it is essential to note that some investigations have indicated that Prox1 overexpression in colorectal cancer may paradoxically facilitate tumor growth, heterogeneous proliferation, and malignant progression. The diverse findings suggest the presence of tissue-specific variations in regulatory pathways and inconsistencies in the influence of identical factors across distinct tumors. Additionally, the mechanism by which Prox1 exerts its effects may be intricately modulated by various signaling pathways beyond the scope of the IFN-γ/STAT1 pathway.

#### 3.2.2 The IFN-γ/JAK/STAT1/p21 pathway

Members of the cyclin-dependent kinases (CDKs) family represent promising candidates for targeted tumor therapies due to their critical role in regulating cell cycle progression through interactions with various factors (113). P21 plays a significant role among the inhibitors of the CDK family. The protein belonging to the CIP/Kip family of CDK inhibitors exerts a negative regulatory effect on CDK activity. Enhanced expression of p21 leads to converting active CDK complexes into inactive forms, inhibiting cell cycle progression (114). Previous studies have demonstrated that the upregulation of p21 expression, mediated by IFN-γ through the activation of STAT1, effectively suppresses tumor cell proliferation, as shown in cell line A431 (oral epidermoid carcinoma) and cell lines HT29 and WiDr (colon adenocarcinoma) (115, 116). Furthermore, in cell line HCT116 (colon adenocarcinoma), it has been proposed that p21 expression regulates cell proliferation and inhibits IFN-γ-mediated apoptosis of tumor cells, which correlates with caspase-3 activity (117). This dual role suggests that p21 may possess opposing effects, inhibiting tumor cell proliferation while simultaneously preventing apoptosis. The combined impact of p21 in gastrointestinal tumors remains to be fully elucidated. If p21 is considered a potential target for tumor treatment, the specific mechanisms by which p21 differentially affects various tumor processes must be further investigated.

#### 3.2.3 The IFN-γ/JAK/STAT1/c-MYC pathway

C-MYC represents a distinctive subset of oncogenes that can promote tumorigenesis without necessitating mutations. This gene is pivotal in regulating the cell cycle, cellular proliferation, genomic instability, and tumorigenesis. Downregulated expression of c-MYC induces genomic instability through mechanisms such as gene amplification, chromosomal rearrangements, and karyotypic instability (118–120). Research has elucidated that c-MYC is regulated by IFN-γ, with regulation potentially occurring through both STAT1-dependent and STAT1-independent mechanisms. This phenomenon has also been investigated explicitly within colorectal cancers, highlighting the intricate interplay between cytokine signaling and oncogene expression. The STAT1-dependent way involves binding STAT1 to conserved GAS elements within the c-MYC promoter, leading to its downregulated expression (121). This result suggests a non-immune mechanism through which IFN-γ may inhibit tumor progression beyond its role in upregulating HLA-DR. Specifically, this pathway appears to disrupt the cell cycle of tumor cells, thereby inhibiting their proliferation and consequently mitigating the progression of gastrointestinal tumors (122).

### 3.3 Pathways inhibit tumor progression by promoting tumor cell death

Tumor progression and metastasis are intricately linked to tumor cells’ “unlimited” proliferation. Therefore, the induction of cell death represents a crucial strategy for inhibiting tumorigenesis. Cell death can be categorized into programmed and non-programmed mechanisms. The programmed forms encompass apoptosis and autophagy, while necrosis is classified as a non-programmed mode of cell death (123, 124). In this context, we provide a comprehensive overview of the downstream factors associated with the IFN-γ/STAT1 pathway in gastrointestinal tumors. These factors play significant roles in promoting tumor cell apoptosis and other forms of cell death, thereby inhibiting the advancement of gastrointestinal malignancies.

#### 3.3.1 The IFN-γ/JAK/STAT1/IFIT2 pathway

IFIT2 is an interferon-stimulating factor characterized by its distinct tripeptide repeat sequence (125). It is encoded by the ISG54 gene, which researchers have identified as significantly upregulated in response to viral infections and interferon treatment. The TPR of IFIT2 induces apoptosis in tumor cells through the mitochondrial pathway, thereby exerting an antitumor effect (126–128). The IFN-γ/STAT1 pathway upregulates the expression of IFIT2, leading to the initiation of apoptosis by modulating the balance between anti- and pro-apoptotic factors, thereby regulating mitochondrial permeability and inducing the death of tumor cells. The most potent inducers of IFIT2 are type I IFNs (IFN-α/β) and type III IFNs (IFN-λ), whereas the most potent inducers of type II IFN (IFN-γ) are much weaker in comparison. However, the expression of IFIT2 induced by the IFN-γ/JAK/STAT1 signaling was significantly higher in colorectal tumor cells compared to IFNα induction (129). This discrepancy may be attributable to the specific cell type under investigation. We hypothesize that IFN-γ may play a more substantial antitumor role in colon tumors, a hypothesis that requires further elucidation. Furthermore, the present study demonstrated that IRF-1 regulates IFIT2 expression, suggesting that IFIT2 functions as a downstream molecule of IRF-1, thereby reinforcing the multi-target regulatory function of IRF-1.

#### 3.3.2 The IFN-γ/JAK/STAT1/IRF-1/downstream antitumor factor pathways

In the second part of the paper, we summarize the role that IRF-1 plays in promoting the progression of gastrointestinal tumors by helping the tumor undergo immune escape. At the same time, related studies have also confirmed the role of IRF-1 in inhibiting tumor progression by inducing apoptosis of tumor cells and activating autophagy (130).

Cysteine proteases(caspases) are a highly conserved class of proteolytic enzymes integral to the cellular apoptotic cascade (131). The activation of these proteases represents a crucial convergence point within the apoptotic pathway, wherein they cleave related cellular proteins, thereby systematically facilitating cellular degradation (132). This mechanism is especially significant in the apoptosis of tumor cells, especially in pancreatic cancers, which exhibit remarkable resistance to prevalent pro-apoptotic therapies such as radiotherapy and chemotherapy (133). Recent investigations have illuminated the potential role of the IFN-γ/JAK/STAT1/IRF-1/procaspase-1 pathway as a pro-apoptotic mechanism in pancreatic cancer (134). The pathway underscores a promising avenue for therapeutic intervention. Notably, among the various regulatory sites present in procaspase-1, an IRF-1 binding site has been identified, with IRF-1 functions as an early responder induced upon activation of the IFN-γ/STAT1 pathway. This activation leads to the consequent upregulation of procaspase-1 expression. Procaspase-1 must undergo activation through cleavage by upstream caspases (such as caspase-8 or caspase-9) or via autocatalytic activity to transform into its active form (135). This active caspase then initiates the cascade of downstream caspases (e.g., caspase-3), which ultimately results in the degradation of critical substrates, thereby contributing to the apoptotic process (136–141). In conclusion, procaspase-1 and IRF-1 play crucial roles within the IFN-γ/STAT1 pathway, promoting the apoptotic death of tumor cells and thus providing viable targets for targeting malignancies.

Autophagy, an evolutionarily conserved process, involves the formation of autophagolysosomes through the fusion of autophagosomes with lysosomes. This process leads to the degradation of cellular contents, thereby providing cells with the necessary energy to survive or remove abnormal substances, such as misfolded proteins and bacteria (142–144). Studies have shown that IFN-γ can inhibit growth and tumor cell death by inducing autophagy in hepatocarcinoma. This process is controlled by IRF-1, which modulates the expression of autophagy-associated proteins. However, the precise molecular mechanism remains to be fully understood (145). Nonetheless, this provides a novel approach for treating gastrointestinal tumors from the perspective of autophagy.

#### 3.3.3 The IFN-γ/JAK/STAT1/surviving pathway

The IFN-γ/STAT1 pathway not only activates downstream factors to exert antitumor effects but also directly interacts with other factors through STAT1 to inhibit tumor progression. Survivin, classified as a member of the inhibitor of apoptosis proteins family, is notably expressed in many malignancies (146). Its primary function lies in inhibiting apoptosis among various apoptotic stimuli (147). The mechanistic basis for survivin’s anti-apoptotic activity is attributed to its capacity to interact directly with pivotal regulatory proteins such as p21 WAF, caspase-3, and caspase-7, effectively obstructing their activation (148). Studies have elucidated a mutually antagonistic relationship between STAT1 and survivin in gastric cancers. Notably, IFN-γ has been found to upregulate the expression of STAT1, leading to the downregulation of survivin expression (149). This regulatory interplay suggests that the IFN-γ/STAT1 pathway may confer a protective mechanism against apoptosis resistance in gastrointestinal tumors by diminishing survivin expression.

## 4 Molecules/medicines targeting the IFN-γ/STAT1 pathway for therapies of gastrointestinal tumors

As discussed above, the IFN-γ/STAT1 pathway plays a dual role in promoting and inhibiting gastrointestinal tumors. This pathway is crucial for tumorigenesis and regression. As researchers continue to investigate the pathway’s targets, an increasing number of molecules and medicines that can interact with it are being explored, hoping that they can provide therapeutic benefits for gastrointestinal tumors. These medicines and molecules have demonstrated the ability to target different pathway components, and their mechanisms differ (**Tables 1** and **2**). Notably, while most of the molecules and medicines have been reported to influence tumor progression through modulation of the pathway, only a few are recognized for their interaction with specific downstream targets, such as PD-L1. Therefore, future research should focus on developing precisely targeted therapeutics that act on the downstream factors of the IFN-γ/STAT1 pathway.

**Table 1:** Molecules/medicines targeting IFN-γ-receptors, JAK1/2, and ISGs.

Molecules/ medicines	Targets	Tumor types	Mechanisms	References
TNF-α	IFN-γ receptors	Liver cancers	Upregulating the IFN-γ-receptor and PD-L1 expression, promoting tumor cell immune escape	(153)
All-transretinoic acid		Colorectal cancers	Upregulating the expression of MGAT3 to support the bisected N-glycosylation and stabilization of the IFN-γRα protein, promoting tumor cell death	(154)
Optineurin			Interacting with AP3D1 to prevent the lysosomal sorting and degradation of palmitoylated IFN-γR1, promoting tumor cell death	(155)
MAGE-C3	IFN-γR1 chains	Esophageal cancers	Enhancing IFN-γR1’s signal transduction capabilities to upregulate the expression of PD-L1, promoting tumor cell immune escape	(29)
YTHDF1		Gastric cancers	Downregulating the expression of IFN-γR1, recruiting DCs, and promoting CD4/8+ T cell infiltration, inhibiting antitumor immune	(151)
Deferoxamine	IFN-γR2 chains	Liver cancers	Alleviating the IFN-γ resistance by upregulating the expression of the IFN-γR2 chains, promoting tumor cell apoptosis	(152)
MTMR2	JAK1/2	Gastric cancers	Upregulating ZEB1 by inactivated JAKs/STAT1/IRF-1 signaling to induce epithelial-mesenchymal transition, promoting tumor cell proliferation and metastasis	(37)
MET		Colorectal cancers	Activating JAKs, promoting the transcription of PD-L1, promoting tumor cell immune escape	(159)
SOCS1		Gastric cancers, liver cancers	Occupying the substrate-binding groove of JAKs, inhibiting their activity and downregulating the expression of PD-L1, inhibiting tumor cell immune escape	(157)
PTPN2	JAK1	Colorectal cancers	Inhibiting the dephosphorylation of JAK1 and STAT1, reducing CD8+ T cell recruitment, inhibiting antitumor immune	(158)
AJUBA			Binding to the FERM domain of JAK1, ultimately downregulating the expression of IFIT2, inhibiting tumor cell death	(129)
HDAC	JAK2	Gastric cancers	Upregulating the expression of JAK2, resulting in increased expression of PD-L1, promoting tumor cell immune escape	(156)
EZH2	PD-L1 gene, IRF-1 gene	Liver cancers	Downregulating the expression of PD-L1 by upregulating the level of H3K27me3 at the promoters of both PD-L1 gene and IRF-1 gene, inhibiting tumor cell immune escape	(32)
Abrine	IDO1, CD47, PD-L1, ect.		Combining with IDO1, regulating the expression of JAK1, STAT1, IDO1, CD47 and PD-L1, inhibiting tumor cell immune escape	(39)
CBX3	PD-L1 gene,STAT1 gene	Colorectal cancers	Binding to the promoters of STAT1 and PD-L1 genes, downregulating the expression of inflammatory factors and PD-L1, inhibiting the chronic inflammatory response-mediated tumorigenesis and tumor cell immune escape	(173)
PD-L1 monoclonal antibodies	PD-L1	Gastric cancers, liver cancers, colorectal cancers, ect.	Specifically binding to PD-L1 expressed by tumor cells, inhibiting tumor cell immune escape	(174–176)

**Table 2:** Molecules/medicines targeting STAT1.

Molecules/medicines	Targets	Tumor types	Mechanisms	References
ERK	STAT1	Esophageal cancers	Promoting the proteasomal degradation of ubiquitinated STAT1, downregulating the expression of IFN-γ, inhibiting tumor cell apoptosis	(163)
HOXC9		Gastric cancers	Downregulating the expression of DAPK and RIG1, inhibiting STAT1 modulation via SHP1, inhibiting tumor cell apoptosis	(172)
PML			Inhibiting STAT1 binding to the CXCL10 promoter, downregulating the expression of CXCL10, suppressing the infiltration of immune cells, inhibiting antitumor immune	(84)
HKDC1		Liver cancers	Interacting with ACTA2 to present cytoplasmic STAT1 to IFN-γR1, promoting STAT1 phosphorylation and nuclear translocation, and upregulating the expression of PD-L1, promoting tumor cell immune escape	(165)
EGF		Esophageal cancers	Activating STAT1, upregulating the expression of apoptosis-related genes, promoting tumor cell apoptosis	(164)
MYC		Liver cancers	Downregulating the expression of STAT1 and PD-L1, inhibiting tumor cell immune escape	(160)
TKIs			Promoting the phosphorylation of STAT1, upregulating the expression of HLA-I in tumor cells, promoting antitumor immune	(169)
Nifurtimox		Pancreatic cancers	Inhibiting the phosphorylation of STAT1 by binding to the Tyr701 site, decreasing CXCR2+ neutrophil recruitment, and alleviating tumor immune burden	(170)
[pIC]PEI		Liver cancers, pancreatic cancers	Promoting the phosphorylation of STAT1, upregulating the expression of CCL2 and MMP13 in pancreatic cancers and the expression of NK2R in liver cancers, promoting antitumor immune	(90, 96)
Statins			Inhibiting the phosphorylation of STAT1, downregulating the expression of PD-L1, inhibiting tumor cell immune escape	(167, 168)
Cyclin G2		Colorectal cancers	Promoting the nuclear translocation of STAT1 by binding to PP2AC, upregulating the expression of CXCL9, promoting antitumor immune	(79)
Bortezomib		Metastatic colorectal cancers	Restoring the downregulated expression of STAT1, upregulating the expression of MHC-I, promoting antitumor immune	(161)
Wedelolactone		Colorectal cancers, liver cancers	Inhibiting the dephosphorylation of STAT1 by TCPTP, promoting antitumor effects	(171)

### 4.1 Molecules/medicines enable therapy by affecting the IFN-γ-receptor

The initial phase of activating the IFN-γ/STAT1 pathway involves the interaction of IFN-γ with its receptor, which includes two IFN-γR1 chains that bind the ligand and two IFN-γR2 chains that facilitate signaling (150). Molecules/medicines can affect the IFN-γ receptor’s expression and signaling function, enabling therapy of gastrointestinal tumors.

Focusing on the expression of the IFN-γ-receptor, YTHDF1 is significantly elevated in gastric tumor tissues, correlating with poor patient prognoses. The knockdown of YTHDF1 upregulates the expression of IFN-γR1 and activates the downstream JAK/STAT1 signaling, which recruits DCs and promotes CD4^+^ and CD8^+^ T cell infiltration. This modulation restores tumor cells’ sensitivity to antitumor immunity, positioning targeted inhibition of YTHDF1 as a novel strategy to enhance immunotherapeutic efficacy in gastric cancers (151). In liver cancers, Deferoxamine (DFO) can upregulate the expression of the IFN-γR2 chain, alleviating the IFN-γ resistance caused by the imbalance in the expression of IFN-γR1 and IFN-γR2 chains and promoting the apoptosis of tumor cells (152). Surprisingly, TNF-α, recognized for its tumoricidal potential, can also promote immune escape and tumor progression in liver cancers by upregulating the IFN-γ-receptor and induction of PD-L1 expression (153). This observation raises the hypothesis that inhibiting classical antitumor factors may be beneficial in treating gastrointestinal tumors. In particular, concurrently administering anti-PD-L1 targeted therapies alongside TNF-α could yield better therapeutic outcomes in suppressing tumor progression.

Turning to the signaling function of the IFN-γ receptor, downstream factors like IRF-2 mentioned above repress the pathway’s activation via negative feedback on the IFN-γ-receptor promoter, downregulating the expression of IFN-γ-induced pro-apoptotic factors (74). MAGE-C3 binds to IFN-γR1 chains in esophageal cancers, enhancing its signal transduction capabilities to upregulate PD-L1 expression. This mechanism facilitates immune escape. It also promotes the epithelial-mesenchymal transition associated with esophageal cancer metastasis, which is closely linked to STAT3 (29). Furthermore, all-trans retinoic acid and optineurin in colorectal cancers have been found to promote colorectal tumor cell death by stabilizing the IFN-γ receptor. All-trans retinoic acid can upregulate the expression of MGAT3, which supports the bisected N-glycosylation and stabilization of the IFN-γRα protein (154). Similarly, optineurin interacts with AP3D1 to prevent the lysosomal sorting and degradation of palmitoylated IFN-γR1, thus preserving the functionality of IFN-γ and MHC-I signaling (155). In conclusion, these factors promise to treat gastrointestinal tumors by maintaining receptor function.

### 4.2 Molecules/medicines enable therapy by affecting JAK1/2

The Janus kinase family, specifically JAK1 and JAK2, are crucial non-receptor tyrosine kinase family members and play a vital role in signal transduction pathways, particularly the IFN-γ/STAT1 pathway. Upon dimerization of the IFN-γ-receptors, JAK1 and JAK2 cross-phosphorylate the receptors, facilitating further phosphorylation events necessary for recruiting STAT1 (150). Thus, the functionality—whether regular or aberrant—of JAK1/2 is essential for modulating the IFN-γ/STAT1 pathway, influencing the progression of various gastrointestinal malignancies. Extensive research has clarified the role of multiple molecules and medicines in modulating JAK1/2 expression and functions, thereby affecting gastrointestinal tumor progression.

Regarding the expression of JAK1/2, histone deacetylase (HDAC) has been shown to promote the activation of the IFN-γ/STAT1 pathway in gastric cancers, resulting in increased expression of PD-L1. Conversely, the inhibition of HDAC leads to a reduction in PD-L1 expression, attributed to the downregulation of JAK2, which subsequently affects the phosphorylation cascade and mitigates immune evasion by gastric tumor cells (156). We did not find any other articles on mediating gastrointestinal tumor progression by affecting JAK expression, which may emphasize the importance of JAK function rather than quantity in pathway signaling.

Turning to the function of JAK1/2, the suppressor of the cytokine signaling (SOCS) family, including SOCS1, acts as a class of negative feedback regulators of JAK/STAT signaling. SOCS1 has been identified as a critical inhibitor of gastric cancers, liver cancers, and other gastrointestinal malignancies by blocking the IFN-γ/STAT1 pathway. Its mechanism involves acting as a pseudo-substrate that occupies the substrate-binding groove of JAK1/2, inhibiting its catalytic activity and downregulating the expression of immune escape-related genes, including PD-L1 (157). Furthermore, SOCS1 displays antitumor properties by limiting the progression of chronic inflammation-induced tumorigenesis (60). On the contrary, research into the IFN-γ/JAK/STAT1/IFIT2 pro-apoptotic pathway has uncovered that AJUBA, with increased expression in colorectal cancers, may induce tumor progression by specifically binding to the FERM domain of JAK1. This interaction disrupts the connection between JAK1 and the IFN-γ-receptor, inhibiting STAT1 phosphorylation and nuclear translocation, ultimately resulting in downregulated expression of IFIT2. These findings imply that AJUBA is a potential diagnostic marker and a novel therapeutic target for targeting IFIT2 in colorectal cancers (129). It is easy to find that both SOCS1 and AJUBA inhibit the phosphorylation function of JAK by binding to the relevant sites on JAK. However, they had utterly opposite effects on different gastrointestinal tumors. The reasons for these differences still need to be continuously explored. Myotubularin-related protein 2 (MTMR2) promotes gastric tumor progression by influencing the IFN-γ/STAT1 pathway. MTMR2 inhibits the signaling cascade, suppressing IRF-1 expression, relieving the inhibitory effects of IRF-1 on ZEB1, and facilitating epithelial-mesenchymal transition in tumor cells. Although the precise molecular mechanisms by which MTMR2 operates remain unspecified, it has been demonstrated that MTMR2 inhibition enhances JAK1/2 phosphorylation, indicating a potential role in modulating the phosphorylation function of these kinases (37). In colorectal cancers, PTPN2 and MET have also been recognized as modulators of JAK1/2 phosphorylation, thereby affecting the signaling of the pathway. PTPN2 acts as a negative regulator by inhibiting the dephosphorylation of JAK1 and STAT1. At the same time, its inhibition enhances pathway activation and promotes tumor infiltration by CD8+ T cells, possibly improving the efficacy of anti-PD-1 therapies (158). In colorectal tumor cells with MET expansion, the upregulation of PD-L1 expression aligns with the activation of the IFN-γ/STAT1 pathway, attributed to the increased phosphorylation of JAK1/2, facilitating STAT1 activation in response to IFN-γ stimulation and promoting the transcription of PD-L1. These findings suggest inhibiting the PD-L1 target and concurrent anti-MET treatment strategies may provide additional clinical benefits (159).

### 4.3 Molecules/medicines enable therapy by affecting STAT1

In the IFN-γ/STAT1 pathway, STAT1 is recruited to specific binding sites following receptor phosphorylation, in which phosphorylation and dimerization mediated by JAKs succeed. This process concludes with the translocation of STAT1 to the nucleus, which regulates the transcription of downstream target genes (150). The expression and function of STAT1 greatly influence gene expression, playing a crucial role in the pathogenesis and progression of gastrointestinal tumors. Furthermore, modulating STAT1 activity with various molecules or medicines may offer additional therapeutic benefits in gastrointestinal tumor treatment.

Focusing on the expression of STAT1, notably, the inhibition of MYC in liver cancer is linked to elevated STAT1 expression, which upregulates PD-L1 and contributes to immune escape by tumor cells. This dynamic suggests that concurrent targeting of MYC and PD-L1 could provide a synergistic approach to liver cancer therapy (160). Furthermore, the proteasome inhibitor bortezomib has been shown to restore the downregulated expression of STAT1 and phosphorylated STAT1 signaling. This restoration promotes the downstream expression of MHC-I in microsatellite-stabilized metastatic colorectal tumors (mCRC), providing a theoretical foundation for innovative immunotherapy combinations (161).

The effects of STAT1 on function can be divided into three parts: phosphorylation, stability, and nuclear translocation. Here, we found that molecules or medicines focused on affecting the phosphorylation of STAT1 in the pathway to control the progression of gastrointestinal tumors more often than not. The fact that molecules or medicines affect STAT1 phosphorylation in different ways within different digestive organs may explain the differences in clinical benefit of treating different gastrointestinal tumors with the same molecule or drug.

Regarding the phosphorylation of STAT1 in esophageal cancers, patients exhibiting low ERK and elevated STAT1 expression levels demonstrate improved survival outcomes (162). This phenomenon can be attributed to ERK’s capacity to enhance the proteasomal degradation of ubiquitinated STAT1 and downregulate the expression of IFN-γ, inhibiting the pro-apoptotic effects (163). Consequently, targeted inhibition of ERK represents a novel therapeutic strategy for esophageal cancers, potentially realized through the activation of the IFN-γ/STAT1 pathway. Moreover, epidermal growth factor (EGF) has also been reported to inhibit tumor cell proliferation in esophageal cancers via STAT1 activation, paralleling the signaling of IFN-γ that induces apoptosis in esophageal tumor cells. We speculate that EGF likely upregulates the expression of apoptosis-related genes downstream of the IFN-γ/STAT1 pathway, thereby facilitating the apoptosis of esophageal tumor cells. Intriguingly, findings suggest that the pro-apoptotic effects induced by IFN-γ are selectively exerted in tumors, while normal esophageal epithelial cells experience differentiation-promoting effects in response to IFN-γ (164). This observation may be due to phenotypic cell differences and underlying genetic alterations. In conclusion, enhancing EGF signaling might also represent a promising therapeutic avenue for esophageal cancers. In liver cancer, HKDC1 and atorvastatin have been identified as modulators of STAT1, influencing downstream PD-L1 expression and consequently affecting tumor progression. The aberrant expression of HKDC1 has been associated with patient survival outcomes, with its role in liver cancer progression involving upregulating the expression of PD-L1 through STAT1 activation in tumor cells. This phenomenon can be attributed to the interaction of HKDC with the cytoskeletal protein ACTA2, facilitating the upregulation of PD-L1 expression. This process includes the phosphorylation of STAT1 upon engaging with IFN-γR1 in the cytoplasm, followed by nuclear translocation. Consequently, the strategic inhibition of HKDC1 and the administration of anti-PD-L1 therapies are anticipated to enhance therapeutic efficacy in treating liver cancer (165, 166). Atorvastatin, primarily recognized as a cholesterol-lowering agent, has emerged in recent studies as a potential modulator of tumor progression, particularly in liver cancer. This modulation occurs through the inhibition of STAT1 phosphorylation, which subsequently impacts downstream PD-L1 expression via the IFN-γ/STAT1 pathway (167). Similarly, research in pancreatic cancers has demonstrated that statins not only reduce PD-L1 expression but also alleviate the gemcitabine-induced phosphorylation of STAT1 (168). These findings underscore the importance of considering the interplay between statins, tumor immune responses, and the effectiveness of tumor therapies in patients requiring gastrointestinal tumor treatments. Furthermore, tyrosine kinase inhibitors (TKIs), such as regorafenib, have been shown to enhance tumor antigen presentation and stimulate antitumor immunity through the activation and phosphorylation of STAT1 by IFN-γ, promoting HLA-I expression in liver cancer cells (169). Additionally, [pIC]PEI, which aids in cytoplasmic delivery of polyinosinic-polycytidylic acid and Nifurtimox, has promising therapeutic effects in pancreatic cancers by modulating immune cell response within the IFN-γ/STAT1 pathway. Specifically, [pIC]PEI promotes STAT1 phosphorylation, upregulating the expression of CCL2 and MMP13, which promotes macrophage polarization and T-cell activation, strengthening antitumor immunity (90). In liver cancer, [pIC] also activates the IFN-γ/STAT1 pathway, resulting in significant antitumor effects. However, this mechanism is also associated with the activation of NK2R (96), highlighting the variability in action mechanisms of identical molecules across different gastrointestinal malignancies. Conversely, Nifurtimox reduces STAT1 phosphorylation by binding to the Tyr701 site on STAT1, a mechanism that decreases CXCR2+ neutrophil recruitment and alleviates tumor burden in pancreatic cancers, providing a novel avenue for therapeutic intervention (170).In colorectal cancers, Wedelolactone has been observed to enhance the IFN-γ/STAT1 signaling, reinforcing its antitumor effects by inhibiting the dephosphorylation of STAT1 by T-cell protein tyrosine phosphatase (TCPTP). The phenomenon is also documented in liver cancer cells (171). Besides, a study on gastric cancers has revealed that HOXC9 contributes to tumor progression by inhibiting STAT1 signaling. HOXC9 downregulates the expression of DAPK and RIG1, inhibiting STAT1 modulation via SHP1. Although this study does not describe the specific effect of HOXC9 on the phosphorylation of STAT1, targeted inhibition of HOXC9 in gastric tumor cells may enhance the IFN-γ/STAT1 signaling, thereby overcoming tumor-induced resistance to IFN-γ-mediated apoptosis (172).

Turning to the stability and nuclear translocation of STAT1, PML, a recognized tumor suppressor protein, is reduced in advanced gastric cancers but further contributes to creating an immune-enhanced tumor microenvironment. Mechanistically, the loss of PML increases STAT1 binding to the CXCL10 promoter, likely associated with a PML-regulated region on the promoter, thus upregulating CXCL10 expression in tumor cells and aiding the infiltration of immune cells (84). In colorectal cancers, the expression of Cyclin G2 is significantly upregulated in macrophages in response to IFN-γ, leading to increased secretion of CXCL9, enhanced CTL chemotaxis, and inhibition of angiogenesis. This upregulation also facilitates the nuclear translocation of STAT1, boosting CXCL9 mRNA expression, which results from G2’s ability to counteract the inhibitory nuclear translocation induced by the binding of PP2AC to STAT1 through competitive interactions (79). Targeted upregulating G2 expression within the tumor microenvironment of colorectal cancers may hold promise for advancing immunotherapeutic strategies.

### 4.4 Molecules/medicines enable therapy by affecting ISGs

STAT1 undergoes phosphorylation to form homodimers and subsequently translocates to the nucleus, where it plays a pivotal role in regulating gene expression by binding to the γ-activation site (GAS element) within the promoters of ISGs. This mechanism significantly impacts cellular phenotype and physiological functions (150). Although several therapeutic molecules and medicines have been identified that target ISGs to influence gastrointestinal tumor progression, these interventions remain insufficient compared to those addressing upstream components of the pathway. Therefore, continued exploration of molecules and medicines targeting ISGs is essential for achieving more precise clinical treatments. Based on their mechanisms of action, these agents can be categorized into two parts: those that influence the transcriptional expression of ISGs and those that affect the post-translational products of ISGs.

Focusing on the transcriptional regulation of ISGs, recent findings in liver cancer indicate that the epigenetic modifier EZH2 can suppress PD-L1 expression by upregulating the level of H3K27me3 at the promoters of both the CD274 gene (which encodes PD-L1) and the IRF-1 gene. Notably, this process does not interfere with IFN-γ signaling, thereby providing a novel approach for selectively targeting PD-L1 while maintaining the functional integrity of IFN-γ in fostering tumor regression (32). In colorectal cancers, the epigenetic regulator CBX3 has been shown to inhibit IFN-γ signaling through its binding to the promoters of STAT1 and PD-L1, thereby modulating the chronic inflammatory response-mediated tumorigenesis and mitigating therapeutic resistance (173).

Turning to the post-translational influences of ISGs, considerable advancements have been achieved with PD-L1 monoclonal antibodies, such as atezolizumab, which have demonstrated efficacy in treating gastric, liver, and colorectal tumors across various clinical stages, particularly in combination therapy settings (174–176). Additionally, Abrine, an IDO1 inhibitor, exhibits dual action; it not only complexes directly with IDO1 to inhibit its immunosuppressive role but also regulates the expression of JAK1, STAT1, and IDO1 within the pathway. Moreover, it can downregulate PD-L1 and CD47 expression, thereby enhancing the immune response of immune cells against tumor cells and synergistically enhancing the effects of immunotherapeutic agents (39). However, the current repertoire of molecules and medicines targeting ISG expression is still limited. Therefore, further research is imperative to address this deficiency and provide robust evidence for the potential of downstream molecules as novel therapeutic targets.

## 5 Clinical management of gastrointestinal tumors

In the previous section, we have substantially introduced the potential therapeutic targets of the IFN-γ/STAT1 pathway in gastrointestinal tumors, as well as the molecules and medicines that may have therapeutic effects against different targets. However, it is well known that the process of translating basic research into clinical application is fraught with difficulties. Translating these potential targets into clinical therapeutic options requires a large number of clinical trials to determine the efficacy, safety, and dosage of therapeutic options for these targets. In this section, we summarize the latest therapeutic options for common gastrointestinal tumors, which are largely based on the most recent guidelines published by the National Comprehensive Cancer Network (NCCN). At the same time, we also summarize some of the more well-researched immunotherapy regimens for gastrointestinal tumors in the context of the emerging therapeutic paradigm of immunotherapy. In addition, we also searched clinical treatment trials for gastrointestinal tumors related to the IFN-γ/STAT1 pathway, although the search results were not what we expected.

### 5.1 Standard clinical treatment for common gastrointestinal tumors

#### 5.1.1 Esophageal squamous cell carcinoma

In the realm of clinical management of esophageal neoplasms, squamous cell carcinoma (SCC) of the esophagus constitutes a primary focus due to its prevalence, accounting for approximately 90% of all esophageal malignancies (177, 178). Moreover, the molecular pathways and therapeutic targets discussed herein are predominantly specific to SCC cells of the esophagus, thus delimiting the scope of this discussion to clinical treatment strategies pertinent to this histological subtype.

The selection of an appropriate therapeutic modality is largely contingent upon the tumor’s anatomical location within the esophagus and its staging according to the TNM classification system (177). Surgical resection remains a cornerstone treatment modality, with its application tailored according to the tumor stage and disease extent, utilizing various resection techniques as appropriate. For early-stage lesions, including carcinoma *in situ* (Tis) and T1a tumors, endoscopic interventions are considered appropriate, contingent upon the absence of lymph node metastases. Esophagectomy, along with other therapeutic approaches, constitutes the primary treatment modality for tumors classified as cT1b and low-risk cT2N0 lesions (177). For tumors exhibiting further local progression—including high-risk T2N0 lesions, any N+ tumors, or cT3 or cT4a Nx tumors—multimodal therapeutic strategies are strongly recommended. This approach typically involves surgical intervention following neoadjuvant chemoradiotherapy. The efficacy of this treatment paradigm has been substantiated by findings from the CROSS trial and is endorsed by the NCCN (177, 179, 180). Among other treatment options, radical chemoradiotherapy is indicated for cervical esophageal cancer and locally advanced cases that are inoperable or where surgery is refused. Immunotherapy has proven effective for metastatic or persistent lesions and as adjuvant therapy following the KEYNOTE-590 and CheckMate-577 trials, respectively. The KEYNOTE-590 trial provided detailed evidence that patients with high PD-L1 expression in esophageal squamous cell carcinoma who received pembrolizumab exhibited the most significant survival advantage (177, 181, 182). Other pivotal clinical studies—including ESCORT-1, CheckMate-648, ORIENT-15, JUPITER-06, RATIONALE-306, GEMSTONE-304, and ESCORT-RWS—have also corroborated the primacy of combining chemotherapy with anti-PD-1/PD-L1 immune checkpoint inhibitors as the standard first-line therapeutic approach for advanced esophageal squamous cell carcinoma (183). The CheckMate-577 trial evaluated nivolumab in patients with residual disease following neoadjuvant therapy, revealing an overall survival benefit across the cohort, with patients with squamous cell carcinoma deriving a greater benefit compared to those with adenocarcinoma (177). In a comparative analysis of the KEYNOTE-590 and CheckMate-577 trials, it was observed that nivolumab employed in the CheckMate-577 study conferred a more comprehensive therapeutic benefit that was independent of PD-L1 expression levels. Consequently, the NCCN recommends nivolumab as an adjuvant therapy for all patients exhibiting residual disease following induction treatment and esophagectomy (177, 180, 183–185).

#### 5.1.2 Gastric cancer

In treating gastric tumors, the primary focus is on gastric cancer, especially gastric adenocarcinoma, as it makes up over 95% in histological classification (186). Similar to esophageal squamous cell carcinoma, different pathological stages of gastric cancer require different treatment approaches.

For early gastric cancer (staged at T1a or T1b), endoscopic resection is the preferred treatment option. Other surgical methods (including major or total gastrectomy) are recommended for localized gastric cancer above T1b without distant metastases (T1b-T4a, N0-N+). Specifically, neoadjuvant or perioperative chemotherapy is advised to shrink tumors in resectable gastric cancers that have reached T2 or higher or have positive lymph nodes (186, 187). FOLT (fluorouracil plus folinic acid, oxaliplatin, and doxorubicin) is increasingly becoming the standard choice for perioperative chemotherapy, as the phase II/III FLOT4-AIO trial showed that perioperative FLOT improves survival and 5-year overall survival rates compared to ECF (epirubicin, cisplatin, and fluorouracil) (188). Postoperative adjuvant therapy is further categorized based on whether D2 lymph node dissection was performed. Patients undergoing D2 dissection are more suitable for postoperative chemotherapy, while those with less extensive dissection or high-risk cases (e.g., T3-T4 or N+) are better candidates for postoperative radiotherapy. Preoperative radiochemotherapy is an option based on lower-level evidence and will not be discussed further (186, 187).

For advanced, metastatic, and inoperable gastric cancer, the NCCN emphasizes palliative care. The current first-line treatment is based on chemotherapy, and combining several chemotherapeutic agents, such as fluoropyrimidine and platinum, proves more effective. The decision to use other targeted drugs depends on the molecular phenotype of the cancer cells. In first-line treatment, trastuzumab combined with chemotherapy is recommended for HER2-positive gastric cancers. For HER2-negative gastric cancers, immune checkpoint inhibitors (e.g., nabulizumab) or zolbetuximab-clzb (which requires CLDN18.2 positivity) in combination with chemotherapy are recommended. It is essential to note that, in both HER2-positive and HER2-negative regimens, the use of nab-paclitaxel and pembrolizumab requires testing to confirm a PD-L1 CPS of ≥1 (186, 187). The KEYNOTE-062 trial highlighted ongoing controversy regarding pembrolizumab, as its combination with chemotherapy did not improve overall survival in patients with CPS ≥1 or 10. However, pembrolizumab alone improved overall survival compared to chemotherapy alone in patients with CPS ≥10, although the difference did not reach statistical significance (189). Besides the molecular phenotypes mentioned, The Cancer Genome Atlas (TCGA) classifies tumors into other types, notably those that are highly microsatellite unstable or mismatch repair deficient (MSI-H/dMMR). Treatment regimens for these gastric cancer phenotypes are generally determined independently of PD-L1 expression. Currently, pembrolizumab alone or in combination with other chemotherapeutic agents is recommended as the preferred first-line treatment in category 2A for patients with advanced MSI-H/dMMR gastric cancer. For second-line treatment of advanced, metastatic gastric cancer, options include ramelimumab combined with paclitaxel (186, 187).

#### 5.1.3 Hepatobiliary cancer

Among hepatobiliary tumors, we found more clinical interest in hepatocellular carcinoma and biliary tract cancer. We reviewed the clinically relevant guidelines from the NCCN. Still, we found that the only more systematic guidelines available were the earlier 2021 guidelines, which emphasized hepatocellular carcinoma, and the 2023 guidelines, which emphasized biliary tract cancer.

For the treatment of hepatocellular carcinoma, early-stage cases can be managed with surgical resection or transplantation (190). Criteria for selecting surgical resection in early hepatocellular carcinoma include a Child- Pugh score (a method to assess liver functional reserve that considers serum albumin, bilirubin, prothrombin time, and subjective assessment of encephalopathy and ascites- classified as Child- Pugh A for compensated liver function as class A, and loss of compensated function as classes B and C) of class A, absence of portal hypertension, and no vascular invasion by the tumor. Although these scores of grade A and uncomplicated portal hypertension are not mandatory for surgery (191), the NCCN has shown that patients meeting these criteria tend to have a better prognosis (190). Liver transplantation is also an option for patients with early-stage hepatocellular carcinoma, as it removes both the tumor and underlying cirrhosis. Additionally, bridging therapy can be used to control tumor progression while waiting for a transplant (192). For unresectable, locally advanced hepatocellular carcinoma, ablation therapy is a common local treatment, categorized into thermal, chemical, and cryoablation methods. Ablation is most effective for tumors <3cm that are in an appropriate location away from other organs and major vessels or bile ducts, with the best outcomes in tumors <2cm (190). Besides ablation, Arterially Directed Therapies (ADTs) are also used as localized treatment options. Radiotherapy becomes an alternative when ablation or arterial-guided therapies cannot be performed. For advanced metastatic hepatocellular carcinoma, the current first- line treatment is atezolizumab (a monoclonal antibody targeting PD- L1) combined with bevacizumab (a VEGF inhibitor), which has demonstrated improved prognosis over sorafenib in the IMbrave 150 trial (175, 193) and was approved by the FDA as a first- line treatment in 2020. Sorafenib and lenvatinib are frequently used as alternative first-line options for Child-Pugh A patients. In second-line therapy, regorafenib and cabozantinib are used for patients with Child-Pugh A liver function, and ramucirumab requires an AFP level of ≥400 ng/mL. Immunotherapies, such as nivolumab and pembrolizumab, are also employed as second-line treatments, with PD-L1 expression evaluated before use (190).

For the treatment of biliary tract cancer, surgical resection is often chosen for patients in the early stages of the disease (194). For advanced metastatic biliary tract cancer, the preferred first-line regimen is Durvalumab combined with Gemcitabine and Cisplatin, replacing the previous regimen of only Gemcitabine and Cisplatin. This combination is indicated for all subtypes of biliary tract cancer and has been shown to significantly improve patient survival and objective remission rates (194, 195). Second-line treatments include targeted therapy, immunotherapy, and chemotherapy. The choice of targeted therapy depends on the results of molecular testing. Ivosidenib is recommended for IDH1-positive patients and is suitable for both intrahepatic and extrahepatic cholangiocarcinoma. For patients with FGFR2-positive intrahepatic cholangiocarcinoma, Futibatinib or Pemigatinib are recommended. Dabrafenib, in combination with Trametinib, is recommended for patients with all subtypes of progressive BRAF V600E mutation-positive cholangiocarcinoma. The presence of positive biomarkers, including MSI-H/dMMR or TMB-H, guides the use of Pembrolizumab in immunotherapy. While immunotherapy offers the advantage of durable responses, it has the drawback of a low rate of positive biomarker expression in patients (194). Therefore, targeted therapy and immunotherapy show great promise as second-line treatment options for biliary tract cancers compared with hepatocellular carcinoma.

#### 5.1.4 Pancreatic adenocarcinoma

Among all types of pancreatic cancer, pancreatic adenocarcinoma is the most common and most malignant. It is often detected at a late stage, has a poor prognosis, and there are no highly effective treatments for advanced or metastatic malignant pancreatic cancer. For patients with early pancreatic cancer, surgical resection is an option, and if necessary, adjuvant radiotherapy can be used to shrink the tumor and increase the likelihood of successful surgery. These details will not be discussed further here.

For patients with locally advanced or metastatic pancreatic adenocarcinoma, treatment options are divided into first-line and second-line therapies, which are further tailored based on the patient’s physical status and molecular phenotype. In the first line, the main recommended regimens include FOLFIRINOX (5-FU/leucovorin plus oxaliplatin and irinotecan), modified FOLFIRINOX, and gemcitabine combined with albumin-bound paclitaxel. These are typically indicated for patients with good physical status. The modified regimen differs by reducing the drug dose at any one time and shortening the dosing cycle, which may somewhat decrease adverse effects caused by drug toxicity. Gemcitabine combined with cisplatin is reserved for patients in good physical condition with known BRCA1/2 or PALB2 mutations. Monotherapy with gemcitabine, capecitabine, or 5-fluorouracil is generally preferred as the initial treatment for patients with poor physical status who have locally advanced or metastatic disease. Conversely, patients with good physical status may also receive gemcitabine monotherapy combined with erlotinib, in addition to gemcitabine alone. Targeted therapies and immunotherapies are more appropriate for patients with specific molecular phenotypes. Pabolizumab, larotrectinib, and entrectinib are primarily used for metastatic patients with poor physical status and as subsequent treatments; however, pabolizumab is limited to patients with the MSI-H/dMMR phenotype, while larotrectinib and entrectinib are indicated for patients with positive NTRK gene fusions. Most of these regimens can be used in combination with each other as alternative second-line options for patients who do not respond to initial treatments. The combination of 5-fluorouracil analogs with oxaliplatin or irinotecan is more commonly utilized (196).

#### 5.1.5 Colorectal cancer

In the case of colorectal cancer, although we usually discuss the two together, there are some differences in treatment options between the two, especially in the perioperative adjuvant treatment of surgically treatable patients.

The 2024 NCCN clinical guideline on colorectal cancer provides a more systematic overview of treatment options for advanced or metastatic colorectal cancer. For stage I to III colorectal cancers, surgery remains the primary treatment. Adjuvant chemotherapy is mainly recommended for stage II high-risk patients—including those with T4 tumors, bowel perforation, or understaged lymph nodes—as well as stage III patients. The most commonly used regimen is FOLFOX (oxaliplatin + 5-FU/calcium folinate), while CAPEOX (oxaliplatin + capecitabine) is often used as an alternative. Single-agent therapies with 5-FU or capecitabine are more suitable for patients who cannot tolerate oxaliplatin. For advanced or metastatic patients who can tolerate high-intensity treatment and do not have a combination of dMMR/MSI-H or POLE/POLD1 mutations, the first-line treatment options are chemotherapy combined with anti-VEGF (bevacizumab) or anti-EGFR (cetuximab/panitumumab) regimens. The former includes three regimens: FOLFOX, CAPEOX, or FOLFIRI ± bevacizumab, with the FOLFOX/CAPEOX + bevacizumab regimen being the one considered by the NCCN as having class 1 evidence. The latter includes FOLFOX/FOLFIRI + cetuximab/panitumumab regimens, but their use as a class 1 evidence regimen is limited to patients with a genotypic phenotype of RAS/BRAF wild-type and a primary tumor on the left side. For patients who cannot tolerate high-intensity therapy and do not have a combined dMMR/MSI-H or POLE/POLD1 mutation, treatment options include monotherapy with intravenous fluorouracil + calcium folinate, capecitabine, or anti-EGFR monoclonal antibody (limited to left-sided tumors with RAS/BRAF wild-type), or low-intensity combinations such as 5-FU/LV or capecitabine + bevacizumab. Additionally, trastuzumab + anti-HER2 agents are used, but only in cases with HER2 amplification and RAS/BRAF wild-type status. Patients with combined dMMR/MSI-H or POLE/POLD1 mutations may benefit from immune checkpoint inhibitors, which have shown durable responses and low toxicity. These include pembrolizumab and nivolumab ± ipilimumab regimens, though their use is limited to a small subset of mutation-positive patients and is not widely accessible. The second- and third-line regimens, as well as follow-up treatments, are primarily for patients who have progressed after first-line therapy or did not choose the initial regimen, and are not discussed in detail here (197).

Early stages of rectal cancer are treated similarly to most gastrointestinal tumors, with stages one through three often managed surgically. Endoscopic submucosal dissection is indicated for patients with stages T1 and N0. Total mesorectal excision is recommended for locally advanced rectal cancer (T3-T4 or N+), which is the NCCN standard for radical surgery. Patients with stage II to III disease may also receive new adjuvant therapies, including long- and short-course radiotherapy combined with chemotherapy. Long-course radiotherapy can be combined with capecitabine or 5-fluorouracil, while the short-course can be used with CAPEOX or FOLFOX. Combining radiotherapy with chemotherapy can significantly improve patients’ pathological complete remission rates and survival outcomes. For rectal cancer patients with T2N1–2 or T3N0–2 who are suitable for anus-preserving surgery, selective omission of radiotherapy (PROSPECT regimen) may be chosen. This involves neoadjuvant FOLFOX chemotherapy and selective radiotherapy and chemotherapy based on the response, effectively reducing radiotherapy toxicity and improving post-treatment quality of life. However, this regimen requires strict patient screening. Patients achieving complete clinical remission after neoadjuvant therapy may avoid surgery and preserve anal function through a “watch and wait” approach. However, there is a risk of local recurrence, necessitating close follow-up. If recurrence occurs, surgery is still necessary. For advanced or metastatic patients and those with the dMMR/MSI-H molecular phenotype, the treatment plan is generally similar to that for colon cancer and will not be repeated here (198).

### 5.2 Current status of immunotargeted therapy in gastrointestinal tumors

Regarding immune-targeted therapies for gastrointestinal tumors, current options include immune checkpoint inhibitors, antibody-coupled drug therapies, cellular immunotherapies (such as CAR-T cell therapies), tumor vaccines, and immunomodulators, among others. The next section will focus on the first three, as they demonstrate more clinical progress and offer better therapeutic prospects based on the available literature.

#### 5.2.1 Immune checkpoint inhibitors

The use of immune checkpoint inhibitors has been widely promoted, especially PD-1/PD-L1 monoclonal antibodies in esophageal, gastric, and colorectal cancers.

In esophageal cancer, aside from the first-line radiotherapy and chemotherapy combined with anti-PD-L1 treatment, which has proven effective in many phase III trials, trials combining neoadjuvant immunotherapy with chemotherapy are also gradually underway. According to reviews, most of these studies are still in phase I or II. ESCORT-NEO is the first phase III trial to assess neoadjuvant immunotherapy in resectable, locally advanced ESCC, showing that chemotherapy combined with neoadjuvant camrelizumab significantly increased pCR (pathological complete response) compared to chemotherapy alone (from 4.7% to 28%) (199). Although there are encouraging short-term results, long-term survival outcomes require further investigation.

In the treatment of gastric cancer, in addition to the previously mentioned NCCN guidelines, which already include PD-1/PD-L1 monoclonal antibodies combined with platinum-based chemotherapy as part of the recommended first-line treatment, some articles also discuss regional and racial differences in the effectiveness of immunotherapy. Long-term follow-up data from the Checkmate-649 trial suggest that Chinese patients experience a more substantial survival benefit, particularly those with a CPS score of 5 or higher. These differences are probably due to genetic variations between Eastern and Western populations, lifestyle differences, tumor microenvironment diversity, Helicobacter pylori infection status, and differences in healthcare economics. Furthermore, compared to other gastrointestinal tumors, gastric cancer offers a broader range of molecular targets for clinical use. More clinical trials assessing the effectiveness of combination immunotherapy targeting these targets are currently in progress, such as immunotherapy for EBV-positive gastric cancer (200–202).

In immunotherapy regimens for colorectal cancer, although the NCCN mentions using immunotherapy as a first-line treatment for advanced or metastatic colorectal cancer, there are still many limitations, especially for metastatic colon cancer with pMMR/MSS. Due to the presence of numerous immune-inhibitory factors in the tumor microenvironment, immunotherapy outcomes may be disappointing (183, 203). Therefore, for immunotherapy in metastatic colorectal cancer, relying solely on a single immune checkpoint inhibitor may not be enough, and it may be necessary to explore new strategies such as combining two or more immune checkpoint inhibitors with other therapies.

Overall, although immunotherapy regimens using immune checkpoint inhibitors for esophageal, gastric, and colorectal cancers have shown promising therapeutic potential, their development still faces significant challenges. These include a lack of standardization in perioperative immunotherapy due to unclear effects of radiotherapy on different gastrointestinal tumors, unoptimized treatment cycles and sequences, limited survival benefits from combination regimens, tumor microenvironment heterogeneity, and the absence of effective biomarkers, all of which hinder efforts to improve treatment outcomes.

#### 5.2.2 Antibody-drug conjugate therapy

Antibody-drug conjugate therapy involves covalently linking monoclonal antibodies to cytotoxic drugs through chemical linkers, resulting in highly specific targeting and potent cytotoxic effects (204). Although ADC drugs have been extensively developed, most are mainly used for treating hematological tumors. In gastrointestinal tumors, ADC drugs are primarily limited to a small group of target molecules, such as HER2-positive gastrointestinal tumors, especially gastric cancer (204–207). The use of ADCs targeting other molecules in gastrointestinal tumors, such as Claudin18.2 in gastric cancer (208) is gradually being investigated. However, it will still take a significant amount of time before these ADCs can become widely used as standard treatments in clinical practice.

#### 5.2.3 CAR-T cell therapy

Regarding cellular immunotherapy, this section focuses on CAR-T cell therapy, which is the most extensively studied and representative form of CAR-T cell treatment. Its main principle involves using a gene vector carrying chimeric antigen receptors (CAR) to transduce T cells isolated from the patient *in vitro*, thereby converting the patient’s T cells into tumor-specific T cells. These cells are then expanded *in vitro* and reinfused into the patient’s body, allowing them to target and eliminate tumor cells (209–211). Currently, clinical trials of CAR-T therapy for gastrointestinal tumors are also in the development stage. In esophageal and gastric cancers, targets such as HER2 (NCT02713984), EGFR, CEA (NCT02349724), MSLN (NCT03747965), CLDN18.2 (NCT05472857), and NKG2D have shown effective antitumor effects in preclinical and early clinical trials. Additionally, clinical trials targeting CEA and MSLN in pancreatic (NCT05538195, NCT03323944) and colorectal cancers are ongoing. For specific molecular targets in different digestive system tumors, clinical trials targeting the colorectal cancer stem cell marker LGR5 (NCT05759728) in colorectal cancer, EphA2 (NCT05003895) in pancreatic cancer, GPC3 (NCT02395250, NCT03146234), and EpCAM (NCT02729493) in hepatocellular carcinoma, as well as trials targeting MUC1 in pancreatic cancer (NCT03267173) and intrahepatic cholangiocarcinoma (NCT02587689), are all underway, offering broader prospects for the diversified development of CAR-T cell therapy. However, CAR-T cell therapy also faces challenges, including the tumor’s immunosuppressive microenvironment, sensitivity to target selection, and safety concerns, which necessitate ongoing optimization to help CAR-T cells meet the treatment standards outlined in guidelines.

### 5.3 Clinical trial progress of IFN-γ/STAT1 pathway therapy for gastrointestinal tumors

Although we have already provided a detailed discussion of the relevant factors downstream of the IFN-γ/STAT1 pathway from both major directions—promoting and inhibiting tumor growth—and have identified potential drugs or molecules targeting different components within the pathway for targeted therapy, our review of clinical trials related to gastrointestinal tumors on clinicaltrials.gov revealed that, aside from the trials mentioned earlier involving PD-L1 antibodies, there are only a few clinical trials combining drugs with IFN-γ. Most of these trials were completed or terminated early. For instance, a trial evaluating the combination of 5-FU + leucovorin with IFN-γ and bevacizumab in metastatic colorectal cancer (NCT00786643) last updated its data in 2012. This study was not a randomized controlled trial. Although patients showed some positive responses and early response rates, the pathway’s significance was not fully demonstrated because IFN-γ was not included as a variable in the study. There are currently no clinical trials targeting the IFN-γ/STAT1 pathway, either alone or combined with other approaches, nor are there trials using downstream factors of the pathway as biomarkers to guide treatment or assess efficacy. Therefore, further research is necessary to develop pathway-specific clinical treatment regimens for gastrointestinal tumors. Based on current treatment strategies and research trends, combining existing anti-PD-L1 therapies with other upstream or downstream targets of the pathway may be a promising approach.

## 6 Discussion

In this review, we systematically organized the downstream factors of the IFN-γ/STAT1 pathway that promote or inhibit gastrointestinal tumors. Overall, we found that the number of downstream factors regulating tumor immunity is greater than those regulating tumor cell growth or death, which aligns with our understanding of the important role IFN-γ plays in immune regulation. Although many downstream factors exist, this does not mean that all of them can serve as targets for guiding clinical treatment of gastrointestinal tumors. IFN-γ, a traditional immune-inflammatory factor, is widely recognized for its ability to activate the expression of downstream immune-inflammatory genes directly. Consequently, research into new therapeutic targets targeting downstream pathways has increasingly focused on its recently discovered role in promoting tumor immune escape. Therefore, the following discussion primarily focuses on the immune escape-related factors mentioned above, which have greater therapeutic potential.

CD47 and PD-L1 are both crucial immune checkpoints that facilitate tumor immune evasion. However, as summarized earlier, PD-L1 antibodies are used far more frequently than CD47 antibodies in gastrointestinal tumors. Our review of the literature shows that while clinical trials of CD47 monoclonal antibodies are more often registered for solid tumors, there are very few such trials specifically targeting gastrointestinal tumors, and these are mainly conducted in combination with other drugs to compare efficacy against standard treatments (e.g., NCT05002127, currently recruiting) (212). This indicates that treatment strategies targeting CD47 require further development, whether as monotherapy or in combination. Combination therapies have demonstrated better clinical prospects in many tumor studies, especially when paired with PD-L1 antibodies, showing effective anti-tumor activity in colorectal cancer and melanoma models (23, 213–215). In particular, a study on colorectal cancer radiotherapy found that after treatment, cancer cells simultaneously upregulate CD47 and PD-L1 via the ATR-mediated DNA repair signaling pathway (216). Although it remains uncertain whether this involves activation of the IFN-γ/STAT1 pathway, it suggests that combining CD47 and PD-L1 antibodies after colorectal cancer radiotherapy could enhance therapeutic effects and improve patient survival. This may lead to a superior standard radiotherapy approach for colorectal cancer. Future research should investigate the relationship between CD47 and PD-L1 expression and the activation of the IFN-γ/STAT1 pathway in colorectal cancer radiotherapy. If a connection is confirmed, targeting their common upstream regulators, such as STAT1 or IRF-1, could be an exciting research direction. Besides combining immune checkpoint inhibitors, pairing CD47 antibodies with CAR-T cell therapy is also being developed (217). Increasing evidence indicates that CD47-targeted therapy is emerging as a promising new form of immunotherapy.

HHLA2 is also an immune checkpoint, although it has not received as much attention as the previously mentioned CD47 and PD-L1. However, research into its role in gastrointestinal tumor treatment has made rapid progress. Compared to immune checkpoint blockade therapy, more research has focused on HHLA2 as a prognostic biomarker for gastrointestinal tumors. Multiple studies have demonstrated that HHLA2 expression is significantly associated with a poor prognosis in patients with intrahepatic cholangiocarcinoma (218, 219). The expression frequency of HHLA2 is higher than that of PD-L1, making it a potential therapeutic target for intrahepatic cholangiocarcinoma, second only to PD-L1 (218). In pancreatic cancer and advanced gastric cancer, HHLA2 expression is associated with a better patient prognosis (220, 221); however, some studies suggest no correlation between HHLA2 expression and prognosis in pancreatic cancer patients (222). This discrepancy may be due to factors such as differences in the cellular materials used in experiments or variations in analytical protocols. Differences in prognostic predictions across various tumors may relate to tumor type, tumor heterogeneity, clinical stage, or methodological differences in the experiments. In summary, further research is necessary to resolve these discrepancies and establish HHLA2 as a reliable biomarker for predicting patient prognosis. Additionally, the regulation of HHLA2 expression by the IFN-γ/STAT1 pathway has only been observed in liver cancer; further investigation is required to explore the potential therapeutic effects of targeting this pathway to regulate HHLA2 expression in other gastrointestinal tumors.

IDO1 plays a crucial role in tryptophan metabolism and immune suppression, suggesting that inhibiting this key factor could effectively control tumor development. However, a review article on the application of IDO1 inhibitors in cancer immunotherapy indicates that studies on IDO1 inhibitors have not produced the expected benefits for cancer patients, regardless of whether they have gastrointestinal tumors. This may be related to factors such as the expression of IDO1 in multiple pathways and the limited efficacy of inhibitors. Nonetheless, the article emphasizes that combining IDO1 inhibitors with other drugs might be a viable treatment strategy targeting this pathway. Specifically, regarding the IFN-γ/STAT1 pathway, the article suggests that simultaneously inhibiting IDO2 could be one approach to targeting IDO1 expressed in this pathway. However, inhibiting IDO2 currently presents certain challenges, though it may serve as a targeted treatment option for the downstream elements of this pathway (223).

IFITM3 and GBP5 both act as downstream factors of the IFN-γ/STAT1 pathway and can serve as cancer biomarkers. IFITM3 is expressed in gastric, liver, pancreatic, and colorectal cancers (224), and it is a poor prognostic marker in pancreatic ductal adenocarcinoma (225). GBP5 functions as a biomarker predicting a good response to immune checkpoint inhibitor therapy in oral cancer (226, 227). However, their roles were previously thought to involve a feedback loop aiding gastrointestinal tumors in immune evasion. Recent findings have updated our understanding of their functions. Notably, the immune escape role of the IFN-γ/STAT1/IFITM3 feedback loop is mediated by FOXP3^+^ Treg cells, while the IFN-γ/STAT1/GBP5/CXCL8 loop involves MDSCs. This contrasts with the tumor cell clearance functions of CD47 and PD-L1, which inhibit the activity of T cells and phagocytes. These differences suggest that, under the same cytokine influences within the tumor microenvironment, the overall immune response depends on the combined effects of various immune cells. This insight suggests that altering the cellular composition in the tumor microenvironment of gastrointestinal tumors may influence treatment outcomes, particularly in tumors with a higher prevalence of immune escape-promoting cells. Such strategies could represent a next-generation approach to immunotherapy.

In addition to immune escape factors regulated by signaling pathways, cytokines linked to tumor growth, metastasis, and death are also being increasingly recognized for their potential clinical roles in gastrointestinal tumors. For example, MUC4, which belongs to the same family as MUC1, is recognized as a biomarker for cancer metastasis in pancreatic and colorectal cancers, and is also associated with prognosis in pancreatic cancer (228, 229). However, whether they can serve as viable targets for precision therapy in gastrointestinal tumors still requires extensive clinical trials for validation. Nonetheless, given the current research focus, using established immune checkpoint inhibitors, such as PD-L1, combined with new immune targets like CD47, along with various immunotherapy regimens—including bispecific antibodies or monoclonal antibodies combined with immunotherapy—represents the most promising approach for achieving precision therapy in gastrointestinal tumors currently.

## 7 Conclusion and future perspectives

This review clarifies the downstream key factors and mechanisms through which the IFN-γ/STAT1 pathway influences tumor progression in various gastrointestinal tumors, exhibiting both promoting and inhibitory effects. Diverse cellular actions, including modulation of tumor immunity, induction of inflammation, and regulation of tumor cell proliferation and apoptosis, mediate these effects. Among the key factors involved, IRF-1 and its downstream factors have shown us their effects in promoting or suppressing gastrointestinal tumors from multiple perspectives. PD-L1 has emerged as a significant therapeutic target in most gastrointestinal malignancies. Recent investigations into downstream factors with promising therapeutic potential have highlighted additional targets for developing innovative therapeutic regimens. However, critical questions remain regarding the activation of pathway activators, which, to date, have only been demonstrated in select gastrointestinal malignancies. It is unclear whether similar activation will occur in other gastrointestinal tumors and whether it will yield similar pro-tumor or antitumor outcomes. The complexities of these mechanisms, particularly their relationship with the extracellular environment and cellular architectures of various digestive organs, warrant further investigation. It is also essential to emphasize that most studies referenced herein were conducted *in vitro* using cultured animal cells, and the translational relevance of these findings to the complex tumor microenvironment of solid tumors *in vivo* remains to be validated.

Regarding molecules and medicines identified that could potentially enhance therapeutic efficacy against gastrointestinal tumors via the IFN-γ/STAT1 pathway, although there are many therapeutic options, we found that a lack of therapeutic targets remains compared to upstream therapeutics. Further research is needed to develop more accurate and targeted therapeutic interventions for various gastrointestinal tumors. Besides, therapeutic options for targeting upstream regulators, including IFN-γ receptors, JAK1/2, and STAT1, remain uncertain. Specifically, questions arise regarding whether their effects on relevant downstream targets might inadvertently influence other downstream molecules, potentially resulting in either facilitative or inhibitory effects on overall outcomes in gastrointestinal tumors. This complexity underscores the need to prioritize research on developing targeted medicines against ISGs. Notably, a novel therapeutic strategy may simultaneously inhibit carcinogenic downstream targets while maintaining the activation of anticarcinogenic upstream targets within this pathway, potentially leading to superior clinical outcomes.

Returning to the current standard treatment protocols in clinical practice, our review of gastrointestinal tumor treatment strategies that have received significant attention reveals that immunotherapy still faces many limitations. For example, the use of PD-L1 antibodies is limited by the mutation status of patient-related genes, and immunotherapy approaches, such as cell-based therapies, are not widely utilized. Additionally, several clinical trials are targeting the IFN-γ/STAT1 pathway for treatment. This highlights the significance of the treatment approach proposed in this article, which targets this specific pathway.

We believe that the immunosuppressive effects of the tumor microenvironment, particularly those related to PD-L1, remain a key focus area in current immunotherapy research. Although anti-PD-L1 monotherapy has shown partial resistance in certain gastrointestinal tumors, combination therapies targeting PD-L1 alongside other therapeutic agents have demonstrated encouraging results. Treatment regimens combining immune checkpoint inhibitors with other targets have consistently demonstrated superior therapeutic potential, such as the combination of antibodies targeting CD47, HHLA2, and PD-L1. These findings suggest that further exploration of the application potential of the IFN-γ/STAT1 pathway in clinical combination therapy strategies for gastrointestinal tumors is necessary.

In summary, we hope that the insights and considerations provided in this review will stimulate further research and open up new avenues of investigation in the critical field of multi-agent combination therapy for anti-tumor immunotherapy.

## Glossary

ACTA2: Actin alpha 2
AhR: Aryl hydrocarbon Receptor
AJUBA: Ajuba LIM protein
AP3D1: Adaptor-related protein complex 3, delta 1 subunit
B7-H1: B7 Homolog 1
CAR-T: Chimeric antigen receptor T-cell
CBX3: Chromobox Homolog 3
CCL2: C-C motif chemokine ligand 2
CD47: Cluster of differentiation 47
CDKs: Cyclin-Dependent Kinases
COX-2: Cyclooxygenase-2
CTLs: Cytotoxic T Lymphocytes
CXCL8/9/10/11: C-X-C motif chemokine ligand 8/9/10/11
CXCR3: C-X-C motif chemokine receptor 3
DAPK1: Death associated protein kinase 1
DCs: Dendritic cells
DFO: Deferoxamine
Duox2: Dual oxidase 2
DuoxA2: Dual oxidase maturation factor 2
EGF: Epidermal Growth Factor
EMT: Epithelial-Mesenchymal Transition
ERK: Extracellular Signal-Regulated Kinase
ESCC: Esophageal Squamous Cell Carcinoma
EZH2: Enhancer of Zeste Homolog 2
FOXP3: Forkhead Box P3
GAS: γ-Activation Site
GBP5: Guanylate Binding Protein 5
HDAC: Histone Deacetylase
HHLA2: HERV-H LTR-Associating 2
HKDC1: Hexokinase Domain Containing 1
HOXC9: Homeobox C9
H3K36me2: Histone H3 Lysine 36 Dimethylation
H3K27me3: Histone H3 Lysine 27 Trimethylation
IDO1: Indoleamine 2,3-Dioxygenase 1
IFI35: Interferon Inducible Protein 35
IFIT2: Interferon Induced Protein with Tetratricopeptide Repeats 2
IFITM3: Interferon Induced Transmembrane Protein 3
IFN-γ: Interferon-Gamma
IFN-γR: Interferon-Gamma Receptor
iNOS: Inducible Nitric Oxide Synthase
IP-10: Interferon Gamma-Induced Protein 10
IRF-1/2/7/9: Interferon Regulatory Factor 1/2/7/9
ISGs: Interferon-Stimulated Genes
JAK: Janus Kinase
KRAS: Kirsten Rat Sarcoma Viral Oncogene Homolog
Kyn: Kynurenine
MAGE-C3: Melanoma Associated Antigen C3
MCP-1: Monocyte Chemoattractant Protein-1
MDSCs: Myeloid-Derived Suppressor Cells
MET: MET Proto-Oncogene
MGAT3: Mannosyl (Alpha-1,3-)-Glycoprotein Beta-1,4-N-Acetylglucosaminyltransferase
MHC-I: Major Histocompatibility Complex Class I
MMP13: Matrix Metallopeptidase 13
MTMR2: Myotubularin Related Protein 2
MUC1/4: Mucin 1/4
MYC: MYC Proto-Oncogene
NCCN: National Comprehensive Cancer Network
NF-κB: Nuclear Factor Kappa B
NK cells: Natural Killer cells
NK2R: Neurokinin 2 Receptor
NKA: Neurokinin A
NLRC5: NLR Family CARD Domain Containing 5
PD-1: Programmed Cell Death 1
PD-L1: Programmed Death-Ligand 1
PI3K: Phosphatidylinositol 3-Kinase
pIC: Polyinosinic-Polycytidylic Acid
PML: Promyelocytic Leukemia Protein
PP2Ac: Protein Phosphatase 2A Catalytic Subunit
Prox1: Prospero Homeobox 1
PTPN2: Protein Tyrosine Phosphatase Non-Receptor Type 2
RIG1: Retinoic Acid Inducible Gene 1
ROS: Reactive Oxygen Species
SCC: Squamous cell carcinoma
SHP1: SH2 Domain Containing Phosphatase 1
SIRPα: Signal Regulatory Protein Alpha
SOCS1: Suppressor Of Cytokine Signaling 1
STAT1: Signal Transducer And Activator Of Transcription 1
TCPTP: T-Cell Protein Tyrosine Phosphatase
T_H1_: T Helper 1
TNF-α: Tumor Necrosis Factor Alpha
TPR: Tetratricopeptide Repeat
Trp: Tryptophan
Tregs: Regulatory T Cells
WHSC1: Wolf-Hirschhorn Syndrome Candidate 1
YTHDF1: YTH N6-Methyladenosine RNA Binding Protein F1
ZEB1: Zinc Finger E-Box Binding Homeobox 1
